# Coalescent Simulation and Paleodistribution Modeling for *Tabebuia rosealba* Do Not Support South American Dry Forest Refugia Hypothesis

**DOI:** 10.1371/journal.pone.0159314

**Published:** 2016-07-26

**Authors:** Warita Alves de Melo, Matheus S. Lima-Ribeiro, Levi Carina Terribile, Rosane G. Collevatti

**Affiliations:** 1 Laboratório de Genética & Biodiversidade, ICB, Universidade Federal de Goiás (UFG), Cx.P. 131, Goiânia, GO, Brasil; 2 Laboratório de Macroecologia, Universidade Federal de Goiás (UFG), Campus Jataí, Jataí, GO, Brasil; University of Colorado, UNITED STATES

## Abstract

Studies based on contemporary plant occurrences and pollen fossil records have proposed that the current disjunct distribution of seasonally dry tropical forests (SDTFs) across South America is the result of fragmentation of a formerly widespread and continuously distributed dry forest during the arid climatic conditions associated with the Last Glacial Maximum (LGM), which is known as the modern-day dry forest refugia hypothesis. We studied the demographic history of *Tabebuia rosealba* (Bignoniaceae) to understand the disjunct geographic distribution of South American SDTFs based on statistical phylogeography and ecological niche modeling (ENM). We specifically tested the dry forest refugia hypothesis; i.e., if the multiple and isolated patches of SDTFs are current climatic relicts of a widespread and continuously distributed dry forest during the LGM. We sampled 235 individuals across 18 populations in Central Brazil and analyzed the polymorphisms at chloroplast (*trnS-trnG*, *psbA-trnH* and *ycf6-trnC* intergenic spacers) and nuclear (ITS nrDNA) genomes. We performed coalescence simulations of alternative hypotheses under demographic expectations from two *a priori* biogeographic hypotheses (1. the Pleistocene Arc hypothesis and, 2. a range shift to Amazon Basin) and other two demographic expectances predicted by ENMs (3. expansion throughout the Neotropical South America, including Amazon Basin, and 4. retraction during the LGM). Phylogenetic analyses based on median-joining network showed haplotype sharing among populations with evidence of incomplete lineage sorting. Coalescent analyses showed smaller effective population sizes for *T*. *roseoalba* during the LGM compared to the present-day. Simulations and ENM also showed that its current spatial pattern of genetic diversity is most likely due to a scenario of range retraction during the LGM instead of the fragmentation from a once extensive and largely contiguous SDTF across South America, not supporting the South American dry forest refugia hypothesis.

## Introduction

Seasonally dry tropical forests (SDTF hereafter) are deciduous and semideciduous forests globally distributed on tropical regions and characterized by pronounced seasonality in rainfall [1]. The SDTFs occur usually scattered among other vegetation types such as wet forests, savannas and woodlands [2], and the transition forest-savanna is determined mainly by soils conditions and fire frequency [3]. SDTFs occur in regions with similar climates of savannas, but restricted to eutrophic and oligotrophic soils, with moderate *pH* and low levels of aluminum [2].

In South America, the SDTFs are distributed in multiple and isolated patches across a "dry diagonal" [4], including the cactus thorn scrub in more arid areas in the Northeastern Brazil (the Caatinga Biome), the savannas of Central Brazil (the Brazilian Cerrado Biome), and the Chaco woodlands across the ‘Misiones’ nucleus in Bolivia. The SDTFs expand still on moister sites at south of Amazon basin, in the closed canopy semi-deciduous Bolivian Chiquitano forest, and dry valleys in the tropical Andes between Colombia and Bolivia (e.g., the ‘Piedmont’ nucleus, see details in [5, 6]). Previous studies based on contemporary plant occurrences and pollen fossil records proposed that the current disjunct distribution of SDTF across South America is the result of recent fragmentation of a formerly more widespread and continuously distributed dry forest during the arid climatic conditions associated with the Last Glacial Maximum (LGM; 5, 6), which is known as the dry forest refugia hypothesis [7,8], also supported by phylogeographic analyses [9].

Specifically, two paleoscenarios were proposed to explain the current disjunct distribution of SDTF in South America, based on the hypothesis of modern-day refugia. Prado and Gibbs [5] proposed the ´Pleistocene Arc Hypothesis´ (PLAH hypothesis hereafter), which suggests an expansion of SDTFs throughout the South America during the dry-cool periods of the Quaternary glaciations, connecting the paths of SDFTs from the ‘dry diagonal’, i.e., the Caatinga nuclei, the fragments in Central, Southeast and Southwest Brazil, the Misiones, Chiquitano and Piedmont nuclei and the other disjunct areas in tropical Andes. As a consequence, the current fragmented and disjunct distribution pattern would be a climatic relict of a once extensive and largely contiguous Pleistocene arc of seasonal woodland formation. In contrast, Pennington *et al*. [6] propose that the SDTFs did not merely form a ‘Pleistocene arc’ around the periphery of the Amazon basin, but expanded its geographical range toward the Amazonian lowlands during glacial periods, especially during the LGM. Subsequently, STDFs retracted their once wider range on Central and Southeast Brazil during warm climates of Holocene, which caused the current disjunct distribution (PPPH hypothesis hereafter). PLAH and PPPH hypotheses propose thus different distribution dynamics for the SDTFs in South America. However, it should be noted that both hypotheses describe a clear scenario of widespread and continuously distributed SDTFs during the LGM, contrasting with its reduced and fragmented distribution nowadays (i.e., the modern-day refugia).

Recently, SDTFs has been the focus of many studies due to their conservation status and exposure to different threats (e.g. [10,11]) and also evidence of high phylogenetic (see [12] for a review) and phylogeographic structure [9,13]. However, the response of South American SDTF species to the Quaternary climate changes is barely understood, mainly due to the low number of phylogeographic studies (but see [9,13,14]). Actually, recent evidences show complex and variable distribution dynamics of SDTFs in Pleistocene South America, including contrasting scenarios with the modern-day refugia hypothesis. Pollen records, for instance, indicate that current climatic and vegetation conditions have been established only after 4,800 yr BP in some regions of Caatinga, in Northwest Brazil [15]. Other important regions of the current SDTF distribution, such as the Bolivian Chiquitano dry forest, have not Pleistocene ages, but instead arose during the Holocene as a consequence of population expansions from parts of southern Amazonia rain forest [7,8]. Studies using ecological niche modeling (ENM) have also showed species-specific responses to the Quaternary climate changes [16,17], do not supporting the generalized dry forest refugia hypothesis as originally proposed (see example in [18]).

Paleodistribution modeling helps the understanding of past species distribution dynamics, and coupled with statistical phylogeography, may provide clues on the demographic history of populations and the microevolutionary processes underlying the spatial structure of genetic diversity [19,20]. In this context, coalescent modeling provides the methodological background to test alternative hypotheses about the demographic history of populations, as well as the time and patterns of lineage divergence (see [21] for a review). Also, coupling coalescent and niche modeling with fossil evidence is valuable to test conflicting hypotheses about the role of Quaternary climate changes on species dynamics, like the SDTFs in South America.

*Tabebuia roseoalba* (Ridl.) Sandwith (Bignoniaceae) is a seasonally dry tropical forest tree with disjunct distribution throughout the SDTFs of South America (see S1 Fig). It occurs since from northeast Brazil, in the Caatinga biome, towards the Misiones nucleus (southwest Brazil, Bolivia), Paraguay and Peru and also, scattered throughout the fragments of SDTFs in Central Brazil. It is a self-incompatible species pollinated by large-sized bees, such as bumblebees and carpenter bees. The small winged seeds are wind-dispersed. Its populations present low density (usually < 1 ind/ha) and are restricted to SDTFs over calcareous rocks or high fertile soils. Specialist species with narrow ecological niches may be restricted to smaller geographic ranges [22], or may be hindered in tracking environmental changes due to restriction in avalilable suitable habitats to occupy, mainly species with low dispersal capability [23]. Because niche conservatism and rapid climate change rates, the species often track suitable habitats during short time rather than evolve and adapt to new environmental conditions (the “Habitat Tracking” hypothesis) [24,25]. Due to its specific soil requirements, we predict that *T*. *rosealba* did not expand its range during the LGM despite favorable climatic conditions, but maintained disjunct distribution throughout the Quaternary. Our prediction contradicts the distribution dynamics of *T*. *impetiginosa*, a co-generic species with wider distribution range in SDTFs that showed larger range at the LGM than at present-day [9].

Here we studied the phylogeography of *T*. *rosealba* to trace the history of SDTFs in South America and tested the dry forest refugia hypothesis concerning specific climatic oscillation during the last glacial cycle. Our analyses followed the framework proposed by Collevatti *et al*. [9,17], which is based on coalescence simulations of alternative demographic hypothesis. Coalescent simulations were performed under demographic expectations from *a priori* PLAH and PPPH hypotheses and other two demographic expectances predicted by ENMs, which include the combined dynamics of both PLAH and PPPH hypotheses and range retraction at the LGM (instead of range expansion, a formerly widespread and continuously distributed SDTFs, as expected by previous hypotheses). Our phylogeographic inferences indicate that the current spatial pattern of genetic diversity of *T*. *roseoalba* is most likely due to a scenario of range retraction with smaller population size during the arid climatic conditions associated to the LGM, therefore, do not support the South American dry forest refugia hypothesis.

## Material and Methods

### Population sampling

**Fig 1:**
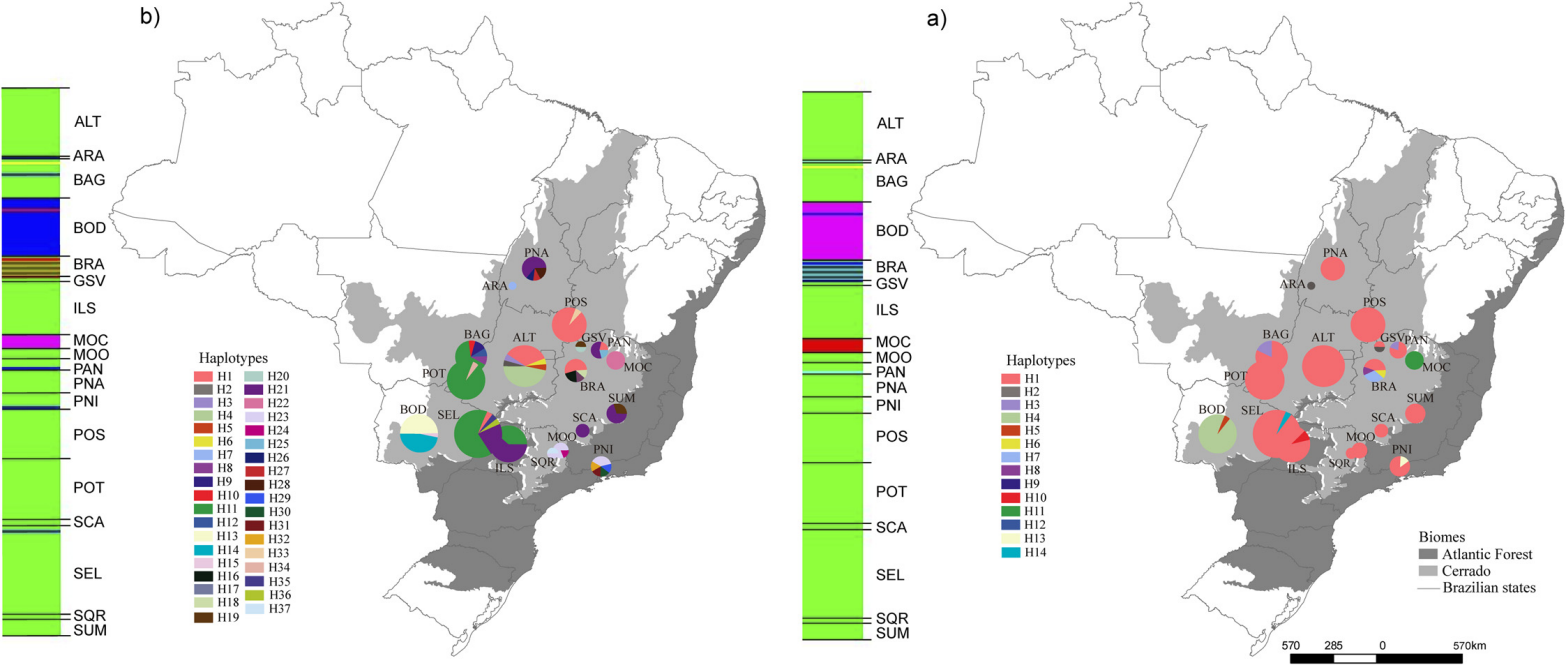
Geographic distribution of haplotypes and Bayesian clustering for (a) ITS and (b) cpDNA, based on the sequencing of 235 individuals of *Tabebuia roseoalba* from 18 populations. Different colors were assigned for each haplotype according to the figure legend. The circle size represents the sample size in each population and the circle sections represent the haplotype frequency in each sampled population. For details on population codes and localities see S1 Table. For BAPS clustering, each color represents an inferred cluster (6 clusters for ITS and 5 for cpDNA). Map modified from the IBGE shape, free available at http://www.ibge.gov.br/home/.

We sampled 18 populations (235 adult individuals) throughout the geographic distribution of *T*. *rosealba* in Brazil during 2011, mainly in SDTFs across Cerrado, Pantanal and Mata Atlântica biomes (Fig 1; see also S1 Table). Because of the high level of anthropic disturbs in the Brazilian SDTFs, some regions presented limited amount of living individuals, resulting in different sample sizes among populations (Table 1). Sampling was not performed in conservation units or private areas and thus did not require any license. The study does not involve endangered or protected species. Vouchers were compared to herbarium material from the Federal University of Goiás (Universidade Federal de Goiás, exsiccate UFG 17962) in Goiânia. Individuals were mapped and expanded leaves or cambium of adult trees was sampled for DNA extraction.

**Table 1:** Genetic diversity and demographic parameters for the 18 populations of *T*. *roseoalba*, for combined cpDNA data and ITS nrDNA. Demographic parameters were estimated based on concatenated data.

Pop	N	cpDNA			ITS			Concatenated					
		*k*	*h*	*π (SD)*	*k*	*h*	*π (SD)*	*θ*	*θ 95%CI*	*g*	*g 95%CI*	*Ne*	*Ne 95%CI*
**ALT**	29	6	0.645	0.0005 (0.0004)	1	0.000	0.0000	0.00051	0.00036–0.00082	576.97	-361.48–937.98	817.31	576.92–1314.10
**ARA**	1	1	0.000	0.0000	1	0.000	0.0000	-	-	-	-	-	-
**BAG**	17	5	0.507	0.0120 (0.0063)	2	0.220	0.0202 (0.0109)	0.00075	0.00025–0.00175	236.66	-431.25–929.49	1201.92	400.64–2804.49
**BOD**	25	3	0.560	0.0026 (0.0015)	2	0.080	0.0050 (0.0031)	0.00029	0.00007–0.00119	-383.94	-479.49–904.57	464.74	112.18–1907.05
**BRA**	9	4	0.694	0.0174 (0.0096)	4	0.750	0.0408 (0.0226)	0.00154	0.00065–0.00538	30.27	-399.60–957.21	2467.95	1041.67–8621.79
**GSV**	2	2	1.000	0.0331 (0.0335)	2	1.000	0.0766 (0.0776)	-	-	-	-	-	-
**ILS**	23	2	0.498	0.0007 (0.0005)	2	0.166	0.0003 (0.0005)	0.00029	0.00005–0.00076	664.97	-460.19–953.45	464.74	80.13–1217.95
**MOC**	6	1	0.000	0.0000	1	0.000	0.0000	0.00004	0.00001–0.00051	-370.44	-454.17–921.94	64.10	16.03–817.31
**MOO**	4	2	0.500	0.0003 (0.0004)	1	0.000	0.0000	-	-	-	-	-	-
**PAN**	5	3	0.700	0.0028 (0.0019)	2	0.400	0.0234 (0.0149)	3.07735	0.01876–8.15501	295.45	-469.47–661.41	493165.6	30064.10–1306892.3
**PNA**	10	4	0.644	0.0005 (0.0005)	1	0.000	0.0000	0.00013	0.00005–0.00131	856.73	-352.38–965.75	208.33	80.13–2099.36
**PNI**	7	5	0.857	0.0036 (0.0022)	2	0.285	0.0012 (0.0012)	0.00171	0.00064–0.00471	727.87	-370.38–955.13	2740.38	1025.64–7548.08
**POS**	21	2	0.095	0.0001 (0.0001)	1	0.000	0.0000	0.00007	0.000001–0.00028	820.65	-415.66–944.12	112.18	1.60–448.72
**POT**	26	2	0.076	0.0001 (0.0001)	1	0.000	0.0000	0.00002	0.00001–0.00052	273.09	-426.80–925.69	32.05	16.02–833.33
**SCA**	3	1	0.000	0.0000	1	0.000	0.0000	-	-	-	-	-	-
**SEL**	38	5	0.486	0.0008 (0.0006)	2	0.052	0.0002 (0.0004)	0.00107	0.00029–0.00237	456.56	-370.74–931.81	1714.74	464.74–3798.08
**SRQ**	2	2	1.000	0.0013 (0.0016)	1	0.000	0.0000	-	-	-	-	-	-
**SUM**	7	2	0.476	0.0003 (0.0004)	1	0.000	0.0000	0.00017	0.00001–0.00072	-348.76	-455.59–916.20	272.44	16.03–1153.85
**Overall**	235	37	0.839	0.0061 (0.0031)	14	0.336	0.0167(0.0086)	0.01299	0.00837–0.01850	-99.010	-204.651–16.713	20817.31	13413.46–29647.44
**Mean**	13.1	2.9	0.485	0.0042	1.5	0.164	0.0093	-	-	-	-	-	-
**SD**	11.2	1.6	0.319	0.0086	0.8	0.279	0.0202	-	-	-	-	-	-

N–sample size; *k*—number of haplotypes; *h*–haplotype diversity; π—nucleotide diversity; SD–standard deviation; *θ*—coalescent parameter; *g*–exponential growth parameter. 95% IC is the credibility interval at 95%. All *g* values did not differ from zero (95% credibility intervals include zero).

### Sequencing analyses

The DNA from leaves and cambium was extracted following the standard CTAB procedure [26]. We sequenced three intergenic spacers of chloroplast DNA (cpDNA): *psbA-trnH trnC*-*ycf6* and *trnS*-*trnG* [27], and the nrDNA ITS1 + 5.8S + ITS2 (primers 75 and 92) [28]. Fragments were amplified by PCR in a 20 μl volume containing 9.0 ng of template DNA, 250 μM of each dNTP, 1.5 μM of each primer, 1.5 unit Taq DNA polymerase (Phoneutria, BR), 1X reaction buffer (10 mMTris-HCl, pH 8.3, 50 mMKCl, 1.5 mM MgCl_2_), 250 μg of BSA. For ITS, we added 2.0μl of DMSO 50%. Amplifications were performed using a GeneAmp PCR System 9700 (Applied Biosystems, CA) with the following conditions: 94°C for 3 min (1 cycle); 94°C for 1 min, annealing temperature for 1 min (50°C for ITS and *psbA-trnH*, 52°C for *trnC-ycf6*, 56°C for *trnS-trnG2S*), 72°C for 1 min (30 cycles); and 72°C for 10 min (1 cycle). PCR products were sequenced on a GS 3500 genetic analyzer (Applied Biosystems, CA) using the BigDye^®^ Terminator v3.1 Cycle Sequencing Kit (Applied Biosystems), according to manufacturer’s instructions. All fragments were sequenced in forward and reverse directions.

Sequences were analyzed and edited to obtain the consensus using the software SeqScape v3.0 (Applied Biosystems, CA) and final alignment was obtained using the software ClustalΩ [29]. Polymorphisms at mononucleotide microsatellites were excluded due to ambiguous alignment and to higher mutation rates. Long indels were coded as one evolutionary event (one character) and each base pair were equally weighted before analysis. For statistical analyses, the sequences of the three chloroplast regions were concatenated.

### Genetic diversity and population structure

Genetic diversity for each population and overall populations was estimated based on nucleotide (π) and haplotype (*h*) diversities [30] using the software ArlequinVer 3.11 [31]. To understand the relationships among haplotypes we inferred intraspecific phylogeny for chloroplast and ITS data using median-joining network analysis implemented in the software Network 4.6.1.0 [32].

The hypothesis of population differentiation was tested and *F*_*ST*_ was estimated based on an analysis of molecular variance (AMOVA) [33] using the software ArlequinVer 3.11 [31]. Population ARA was not used in this analysis because we could sample only one individual. We also analyzed population structure using Bayesian clustering implemented in the software BAPS v5.3 [34]. We performed an individual level mixture analysis with ‘not fixed number of clusters (K)’ with an upper limit of K = 18.

### Demographic history and time to most recent common ancestor

Coalescent model [35] was used to better understand the demographic history of the species and estimate time to most recent common ancestor (TMRCA). Chloroplast and nrDNA ITS data were combined in one analysis, but separate priors were given for each partition. No evidences of heterozygous individuals were found when sequences were analyzed using SeqScape v2.6 (Applied Biosystems, CA). Thus, recombination was neglected in all coalescent analyses. To set the priors, evolutionary model selection for both chloroplast and ITS regions was performed using Akaike Information Criterion, implemented in the software jModelTest2 [36]. For chloroplast regions, the model TVM+G was selected (-lnL = 3065.1841), with gamma shape equal to 0.0170. For ITS, the evolutionary model TIM2+G was selected (-lnL = 1191.6100) with gamma shape equal to 0.0940.

To trace the dynamics in effective population size and historical genetic connectivity we estimated the parameters *θ* = 2*μN*_*e*_ (mutation parameter, *θ* = 4*μN*_*e*_ for diploid genome), *g* (exponential growth rate), where *θ*_*t*_ = *θ*_*now*_exp(-*gtμ*) and *t* is time in mutational unit, and M = 2*N*_*e*_*m*/*θ* (immigration rate, M = 4*N*_*e*_*m*/*θ* for diploid genome). Estimations were based on a Bayesian modeling using Markov Chain Monte Carlo (MCMC) approach [37] implemented in Lamarc 2.1.9 software [38]. The analyses were run with 20 initial chains of 10,000 steps and three final chains of 100,000 steps. The chains were sampled every 100 steps. We used the default settings for the initial estimate of theta. The program was run four times to certify for convergence and validate the analyses using Tracer v1.5 [39] and combined results were then generated. Results were considered when ESS ≥ 200 (effective sample size) and when marginal posterior probability densities were unimodal and converged among runs. The effective population size was estimated from the mutation parameter *θ* using a generation time of 12 years (based on flowering time on permanent plots; RG Collevatti, unpublished data) and the same mutation rate used to *T*. *impetiginosa* [9] and *T*. *aurea* [40]. These analyses were performed only for populations with sample size higher than 4 individuals (13 populations).

We also performed an Extended Bayesian Skyline Plot (EBSP) analysis [41] implemented in BEAST 1.8.2 [42] which calculates the effective population size (*Ne*) through time to better understand changes in population size, combining data from different partitions. We used the substitution models reported above and the relaxed molecular clock model (uncorrelated lognormal) for both chloroplast and ITS. Mutation rates for both chloroplast and ITS regions were the same used for a taxonomic related species [9,40]. Four independent analyses were run for 30 million generations. Convergence and stationarity were checked, and the independent runs were combined using the software Tracer v1.6. Results were considered when ESS ≥ 200. Finally, the hypothesis that the current pattern of haplotype diversity and distribution was caused by contraction of an ancient widely distributed population was tested under the assumption of a bottleneck followed by a sudden expansion using Fu’ neutrality test [43] using the software ArlequinVer 3.11 [31].

TMRCA was estimated based on Bayesian coalescent analysis implemented in the software BEAST 1.8.2 [42]. For both chloroplast and ITS, a relaxed molecular clock (uncorrelated lognormal) was assumed. The ucld.stdev parameter (standard deviation of the uncorrelated lognormal relaxed clock) and the coefficient of variation were inspected for among branch rate heterogeneity within the data. When ucld.stdev is abutting 0 there is no variation in rates among branches and a strict molecular clock cannot be reject. In all runs the ucld.stdev was greater than 0.5 and the coefficient of variation frequency histogram viewed in Tracer abutted against zero (~0.5 to 3.0) showing heterogeneity among branches. We assumed population expansion, based on the Extended Bayesian Skyline Plot (EBSP) analysis [44]. Prior *N*_*e*_ was set to assume a lower bound from zero to infinity upper bound with exponential distribution. Four independent analyses were run for 30 million generations. Convergence and stationarity were checked and the independent runs were combined using the software Tracer v1.6 [39]. We also ran an empty alignment (sampling only from priors) to verify the sensitivity of results to the given priors. The analysis showed that our data is informative because posterior values (e.g. posterior probability) were different from those obtained from empty alignment.

### Setting-up demographic hypotheses

#### Hindcasting species distribution

Occurrence records of *T*. *roseoalba* across Neotropics (S1 Fig, S2 Table) were obtained from GBIF (Global Biodiversity Information Facility http://www.gbif.org/). All records were examined for probable errors and duplicates, and the nomenclature was checked for synonymies. The records were mapped in a grid of cells of 0.5° x 0.5° (longitude x latitude) encompassing the Neotropical region to generate a matrix of 33 presences (cells with occurrence records, S2 Table) used for distribution modeling (see below).

We also generate environmental layers as predictors for ENMs using five bioclimatic variables (annual mean temperature, mean diurnal range, isothermality—mean diurnal range/temperature annual range, precipitation of wettest month, and precipitation of driest month) and subsoil pH (30–100 cm). These five bioclimatic variables present low multicollinearity and were selected by factorial analysis with Varimax rotation from 19 bioclimatic variables obtained in the EcoClimate database (www.ecoclimate.org) [45]. The climate predictors present 0.5° of spatial resolution and were obtained for LGM (21 ka), mid-Holocene (6 ka) and pre-industrial (expressing the current climate) periods, using simulations from five atmosphere-ocean general circulation models (AOGCM): CCSM, CNRM, MIROC, MPI and MRI (S3 Table). Subsoil pH (30–100 cm) was obtained from Harmonized World Soil Database version 1.1 [46]. We assumed subsoil pH to be constant throughout the time (from LGM to pre-industrial) and used in ENMs as a “constraint variable” to better model the environmental preferences of *T*. *roseoalba*. Because the SDTF species respond to soil conditions, omitting pH variable from ENMs would predict unrealistically wider potential distributions across present and past climatic scenarios.

The distribution of *T*. *roseoalba* was modeled using 12 methods encompassing both presence-only and presence-absence algorithms (S4 Table). Because real absence data is not available for *T*. *roseoalba*, we randomly selected pseudo-absences throughout the Neotropical grid cells (excepting cells with presences) keeping prevalence equal to 0.5, to calibrate the ENMs based on presence-absence observations. This approach was based on recent studies suggesting that the extent of the geographical region in which the pseudo-absence points are taken have important influences for prediction and performance of ENMs [47,48]. Thus, selecting pseudo-absences throughout the distribution of *T*. *roseoalba* (i.e., the Neotropical region) essentially represents a compromise between generating models that do not generalize well, not produce over predictions of distribution areas that ignore important spatial structure associated with finer scale environmental gradients [48].

The distribution of the *T*. *roseoalba* was first modeled for current (i.e., pre-industrial) climate and then projected onto LGM (21 ka) and mid-Holocene (6 ka) palaeoclimatic conditions. All ENMs used were ran in the integrated computational platform BIOENSEMBLES [49] following the ensemble approach [50]. The procedures for modeling using the ensemble approach were extensively discussed elsewhere [49,9] and just a brief description will be presented below. For each species distribution model, the occurrence points were randomly partitioned into two subsets (training and testing) comprising 75% and 25% of dataset, respectively, and this procedure was repeated 50 times, randomly selecting different combinations of points in the training/testing datasets. Initial models were evaluated by True Skill Statistics (TSS) [51]; models with poor performance (TSS < 0.5) were eliminated (TSS values for all models are provided in S5 Table). Remaining models were combined (an average weighted by TSS value of each model) to generate a frequency of models supporting the occurrence of the species in each cell of Neotropical grid (i.e., consensus maps), for both current and past climatic layers. Next, the predictive maps for LGM, mid-Holocene and present-day were obtained by using the 10^th^ percentile lowest presence threshold; i.e., the 10^th^ percentile of the lowest consensus value linked to a presence record used to build the ENMs.

We applied a hierarchical ANOVA using the predicted suitability from all models (12 ENMs x 5 AOGCMs x 3 Times) as a response variable to disentangle the effects of climate change on species distribution through the time from predictive uncertainties in the potential distribution due to modeling components (i.e. ENMs, AOGCMs). For this, the ENM and AOGCM components were nested into the time component, but crossed by a two-way factorial design within each time period (see [52], for details about hierarchical design).

#### Inferring demographic hypotheses

The 60 palaeodistribution maps (predicted frequencies for the 12 ENMs x 5 AOGCMs) were visually inspected by two of us (RGC and WAM), using a double-blind experimental design, and classified as supporting the alternative scenarios following Collevatti *et al*. [9]: i) the `Pleistocene Arc Hypothesis`, PLAH hypothesis [5], an expansion throughout the Central and Southwest Brazil; ii) the `Amazonian SDF Hypothesis`, PPPH hypothesis [6], a westward range shift, toward the Amazonian Basin; iii) PLAH+PPPH (“Both”), i.e., a prediction for the past occurrence in the regions predicted by both hypotheses, resulting in an expansion throughout the Central and Southwest Brazil and also westward toward the Amazonian Basin; iv) “Range Retraction”, a retraction in geographic range in Central Brazil but without range shift. Although PLAH, PPPH and “Both” scenarios show different distribution dynamics for the SDTF in South America, they are all compatible with the dry forest refugia hypothesis.

#### Demographic history simulation

**Fig 2:**
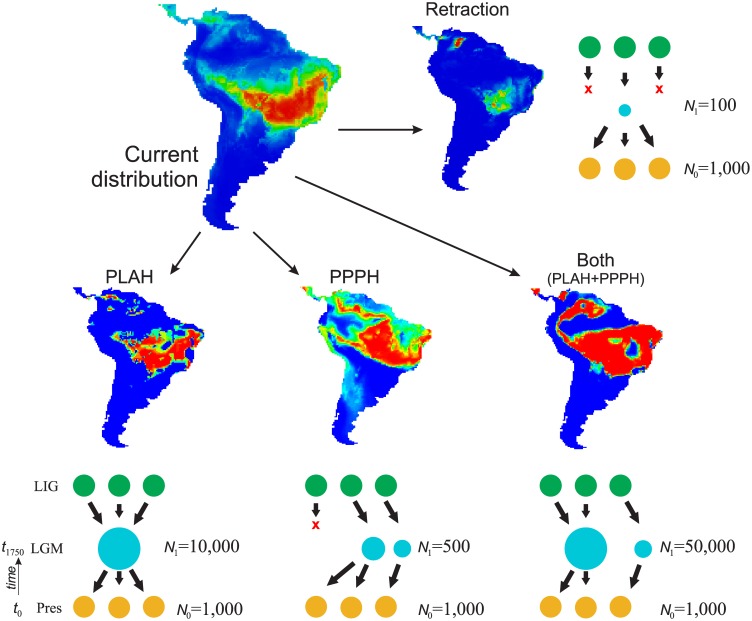
The demographic history scenarios simulated for *T*. *roseoalba* and their geographical representation. The size and location of circle during the LGM indicate demographic population expansion or shrink, and geographic range shift at that time. LIG: last interglacial; LGM: last glacial maximum; Pres: present-day; N0: effective population size at time t0 (present); N1: effective population size at time t1750 (1,750 generations ago). The demographic scenarios correspond to: PLAH, Pleistocene Arc hypothesis; PPPH, the ‘Amazonian SDF’ hypothesis; Both (PLAH+PPPH), i.e., an expansion throughout the Central and Southwest Brazil and also westward toward the Amazonian Basin; Retraction, a retraction in geographic range in Central Brazil.

The demographic history of *T*. *roseoalba* was modeled and simulated based on coalescent analysis [35] implemented in the software ByeSSC [53]. We modeled four demographic scenarios (Fig 2) according to the hypothesis supported by ecological niche modeling and biogeographic hypotheses (PLAH, PPPH, “Both”, or “Range Retraction”), following the framework described in Collevatti *et al*. [9,17]. For each demographic scenario, we run 2,000 independent simulations for each sequence region. Model calibration was based on parameters estimated with Lamarc software and the molecular evolution of the chloroplast non-coding and ITS regions, i.e. the same evolutionary model, sequence size (in base pair) and mutation rates. The number of generations until the LGM (at 21 kyr BP) was calculated using a generation time of 12 years (RG Collevatti unpublished data).

Demographic scenarios were simulated with 18 demes backward from t0 (present) to t1750 generations ago (at the LGM). Population sizes at time t, was calculated from Nt = {ln(N1/N0)/[t]}, where N0 was the same for all scenarios, and N1 shifted among them according to our theoretical expectation (see Fig 2 for details). In BayeSSC negative growth implies population expansion, because coalescent simulations run backward through time. Thus, a negative growth rate implies a population larger now than in the past, and a positive growth, a population smaller now than in the past. Because of the high variation in effective population sizes in *T*. *roseoalba* (see Table 1), we performed simulations with different initial deme sizes, N0 = 100, N0 = 1,000 and N0 = 10,000 for all scenarios. Simulations using N0 = 100 presented all values of haplotype and genetic diversity lower than those observed for *T*. *roseoalba* for all demographic scenarios and N0 = 10,000 retrieved almost all values higher than the observed for *T*. *roseoalba*. Henceforth, we used simulations for N0 = 1,000.

The demographic scenario predicted by ‘Pleistocene Arc Hypothesis’ (PLAH) was simulated using N1 equal to 10,000 and “Both” N1 = 50,000, based on an exponentially positive population growth. The “Amazonian SDF Hypothesis” (PPPH) was simulated using N1 equal to 500 and “Range Retraction” was simulated using the smallest effective population size (N1 = 100), based on a negative population (Fig 2). To simulate migration we considered a finite island model in which all current demes are descendants from lineages originally in deme 1 at t generations ago, meaning that as the tree builds back through time, there is a 0.01/generation chance that each lineage in deme x will migrate to deme 1. We also simulated different values of migration rate but values < 0.01 were not sufficient to show any demographic variation at the time scale we are working and values > 0.1 retrieved equal likelihoods for all models. For ‘Amazonian SDF Hypothesis’ (PPPH) and ‘Range Retraction’ we considered that each lineage in deme x will migrate to deme 1 and than shrink until extinction.

#### Model selection

Simulated alternative models were compared based on the distribution of haplotype and nucleotide diversities in the 2,000 simulations for both chloroplast and ITS sequences. We estimated two-tailed probabilities as twice the number of diversity estimates that were higher than the observed, divided by the number of simulations, so that a high P-value indicates failure to reject the model. We also estimated Akaike Information Criterion (*AIC*) for model choice. The log-likelihood, ln(L), was estimated as the product of the height of the empirical frequency distribution at the observed value of diversity by the maximum height of the distribution. *AIC* (-2Ln(L) + 2K, where K is the number of free parameters, 2 for all models) was transformed into *AIC* weight of evidence (*AICw*), given by exp[-0.5(AIC–AICmin)] [54], from which we obtained *ΔAIC*; i.e. the difference of *AICw* between each model and the best model. Models with *ΔAIC*< 2 were considered as equally plausible to explain the observed pattern [55]. *AICw* was expressed as a relative value among models [54].

### Spatial patterns in genetic diversity

We used spatially explicit analysis to detect spatial patterns in observed genetic diversity in response to late Quaternary climate oscillations, for both cpDNA and ITS. Spatial expansion may lead to gradients in genetic diversity because of allele surfing during the colonization of new areas and “lead trail” expansion (see [56,57] for reviews).

We first tested if differentiation is an effect of isolation by distance [58]. Pairwise linearized *F*_*ST*_ among pairs of populations were estimated for both cpDNA and ITS and correlated with a geographic distance matrix (logarithm) by a Mantel test using ArlequinVer 3.11 [31].

We used quantile regressions to analyze the relationships of climatic suitability and stability through time with genetic diversity [59]. For this, we calculated the difference of ensemble suitability between LGM and present-day as a measure of climate stability through time. Next, we analyzed whether historical changes in species’ geographic range generated a cline spatial pattern in genetic parameters, haplotype (*h*) and nucleotide (π) diversities, due to expansion of climatically suitable conditions. For this, we obtained the distance between each sampled population and the centroid of historical refugium, and then we performed quantile regressions of genetic parameters against this spatial distance.

## Results

### Sequence characterization

Amplification of the chloroplast intergenic spacers *psbA-trnH*, *trnC-ycf6* and *trnS-trnG* generated fragments of 461bp, 698bp, and 747bp, respectively. The combined data presented 1,519bp usable sites (excluding microsatellites and coding indels as one evolutionary step), 157 polymorphic sites and 37 different haplotypes for the 235 individuals of *T*. *roseoalba*. For ITS1 + 5.8S + ITS2 (ITS) we obtained a fragment of 533bp. The final alignment presented 506bp usable sites, 94 polymorphic sites and 14 different haplotypes.

### Genetic diversity and population structure

Chloroplast genome showed higher haplotype diversity (*h* = 0.839) than nuclear ITS (*h* = 0.336), but nucleotide diversity (π = 0.0061, SD = 0.0031) was higher for nuclear genome (π = 0.0167, SD = 0.0086, Table 1). Some populations presented no polymorphism for chloroplast genome (ARA, MOC, SCA) and most populations presented no polymorphism for ITS (ALT, ARA, MOC, MOO, PNA, POS, POT, SCA, SRQ, SUM), even populations with larger sample sizes (Table 1). Populations PNI and PAN had the higher haplotype diversities for chloroplast combined data (excluding population with sample size < 2), and populations BRA and PAN had the highest haplotype diversities for ITS (Table 1).

Nuclear ITS showed one widespread haplotype shared by most populations (Fig 1a) and 13 exclusive haplotypes. Three chloroplast haplotypes (H1, H11 and H21) were widespread and shared by many populations (Fig 1b) but 33 haplotypes were exclusive to only one population. Despite some differences in topology, phylogenetic relationships among chloroplast haplotypes and ITS (S2 Fig) did not match the geographic distribution of lineages.

Analysis of Molecular Variance showed a high differentiation among populations for both chloroplast (*F*_*ST*_ = 0.624, p < 0.001) and ITS regions (*F*_*ST*_ = 0.751, p < 0.001). Pairwise differentiation was high and significantly different from zero for most population pairs for both chloroplast and ITS regions (S6 Table).

Bayesian clustering for cpDNA and ITS was very similar and indicated an optimal partition of 6 groups for ITS and 5 groups for cpDNA (Fig 1a and 1b). ITS cluster 6 was comprised by two individuals from cluster 3 of cpDNA (one from ARA and one from PAN). The Bayesian clustering showed no congruence with population geographical distribution.

### Demographic history and time to most recent common ancestor

**Fig 3:**
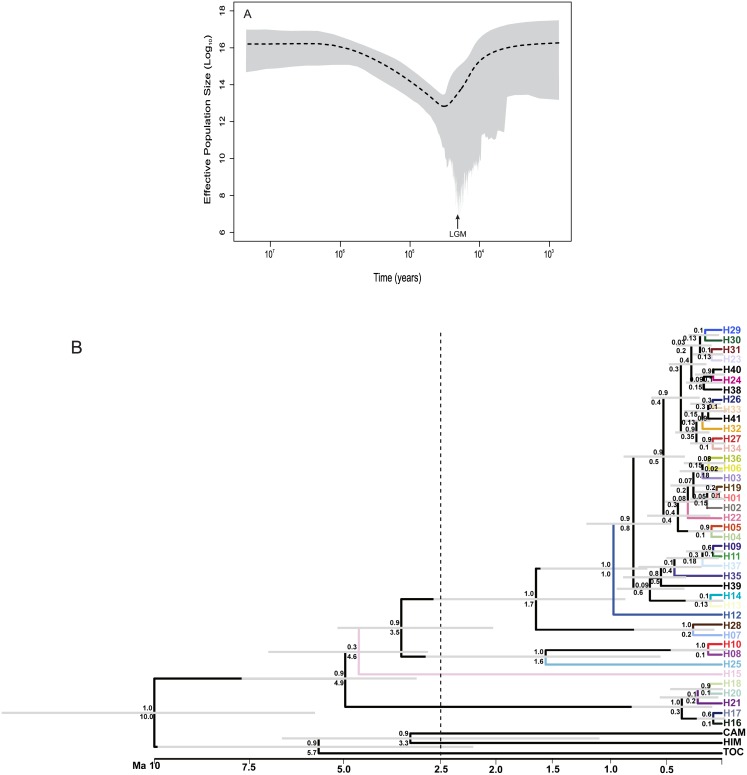
Variation in effective population size through time and relationships and TMRCA (time to most recent common ancestor) of *Tabebuia roseoalba* lineages based on concatenated sequences of cpDNA and ITS nrDNA, from 235 individuals. (**a**) Extended Bayesian Skyline Plot showing effective population retraction at the LGM. (**b**) Coalescent tree showing that most lineage divergences occurred after the Lower Pleistocene. Tip color corresponds to haplotypes described in Fig 1; Gray bar corresponds to 95% Credibility Interval of the mean time to the common ancestor; numbers above the branches are the support to the node (posterior probability); numbers below the branches are the node dating (time to the common ancestor). Time scale is in millions of years (Ma) before present.

Coalescent analysis performed with Lamarc software showed constant population size (Table 1). However, Extended Bayesian Skyline Plot analysis showed a retraction at the LGM followed by an expansion (Fig 3a). We also found low values of mutation parameter *θ* for all populations (Table 1) and overall population (*θ* = 0.01299). Population PAN had a higher mutation parameter (*θ* = 3.07735). Significant effect of population retraction followed by an expansion was found overall populations for both ITS (*FS* = -24.350, p < 0.0001) and chloroplast genome (*FS* = -24.222, p < 0.0001). When populations were analyzed, Fu’s neutrality test was significant for all populations (sample size > 2 and k > 1) for both ITS and cpDNA (all p < 0.05). Using the mutation rate reported above and the generation time of 12 years (RG Collevatti unpublished data) we estimated effective population size *Ne* = 20,817.31 (95% CI: 13413.46–29,647.44). Most populations had high effective population size (Table 1).

Gene flow among all population pairs was negligible (less than 1.0 migrant per generation, S7 and S8 Tables) for all population pairs except PAN that presented high number of migrants per generation, most likely an effect of the high effective population size.

Coalescent analyses performed with BEAST software indicated an ancient time to most recent common ancestor (TMRCA) for *T*. *roseoalba* (Fig 3b), ~4.9 ± 1.9 Ma, which coincides with the coalescence of haplotypes H16, H17, H18, H19 (BRA) and H21 (GSV) from central east Brazil, and all other haplotypes. Most divergences occurred after the Lower Pleistocene, ~1.0 ± 0.6 Myr BP (Fig 3b).

### Setting-up demographic hypotheses

#### Hindcasting species distribution

**Fig 4:**
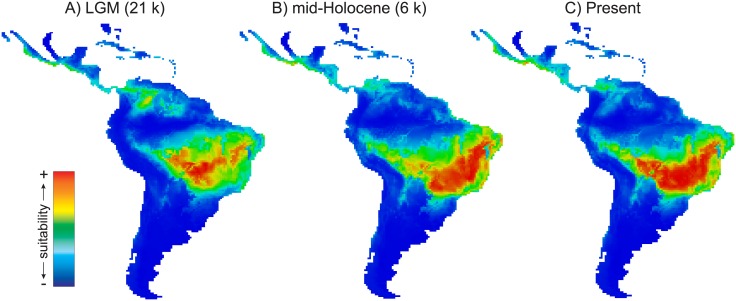
Maps of consensus expressing the ensemble of climatic suitability for *Tabebuia roseoalba*, hence its potential distribution across the Neotropics. (**A**) LGM (21ka), (**B**) mid-Holocene (6 ka), and (**C**) present-day.

The ensemble of ENM predictions reveals that the potential distribution of *T*. *rosealba* at LGM extends mainly over the Central-West Brazil and the Northeast toward the West (Fig 4a). Also, the geographical range was smaller at the LGM than mid-Holocene (Fig 4b) or present-day (Fig 4c, see also S3 Fig). A range shift towards the Northeast and the Southeast was also predicted through the time, mainly from LGM to mid-Holocene and became stable until the present-day (Fig 4, see also S3 Fig).

The analysis of uncertainty using hierarchical ANOVA showed lower proportional variance from time component than modeling method (S9 Table). However, variation was highly structure (S4 Fig) with higher proportion of variation from time component and lower from ENMs and AOGCMs within the geographic range of *T*. *roseoalba*, indicating that the ENMs were able to detect the effects of climate changes on the distribution dynamics of *T*. *roseoalba* through the last glaciation, despite the AOGCM variation (S4 Fig).

#### Inferring demographic hypotheses and model selection

The pattern supporting “Range Retraction” was the most frequent (40%) among the 60 paleodistribution maps. The exact patterns supporting either PLAH (16.7%) or PPPH (28.3%) were less frequent than “Range Retraction” classification. “Both” hypothesis (PLAH+PPPH) was also little supported (15%).

Similarly, the coalescent simulations of alternative demographic hypothesis suggested that “Range Retraction” (a smaller range during the LGM than the present-day, Fig 2) was the most likely scenario to explain the current pattern of genetic diversity of *T*. *roseoalba* compared to other competing hypotheses, using either two-tailed probability or *AICw* criteria (Table 2). Except for cpDNA nucleotide diversity, the scenario PPPH also predicted the observed diversity, but with a slightly lower likelihood than “Range Retraction” (Table 2). The demographic scenario “Both” (PLAH+PPPH) retrieved diversity estimates higher than the currently observed in *T*. *roseoalba* for both haplotype and nucleotide diversity in cpDNA and ITS.

**Table 2:** Comparison of the four demographic models in retrieve the haplotype (*h*) and nucleotide (*π*) diversities observed for *T*. *roseoalba*, obtained from 2,000 simulations using the software BayeSSC.

Diversity index^a^	Models^b^	*P*^c^	Δ*AIC*^d^	*AICw*^e^
*cpDNA*				
*h*	PLAH	0.383	2.963	0.126
	PPPH	0.645	1.606	0.248
	BOTH	0.284	4.033	0.073
	Retraction	0.943	0.000	0.553
*π*	PLAH	0.495	1.862	0.207
	PPPH	0.792	3.107	0.111
	BOTH	0.457	2.421	0.157
	Retraction	0.952	0.000	0.525
ITS				
*h*	PLAH	0.483	0.949	0.246
	PPPH	0.744	0.856	0.258
	BOTH	0.178	2.758	0.099
	Retraction	0.937	0.000	0.396
*π*	PLAH	0.494	2.197	0.145
	PPPH	0.841	0.611	0.321
	BOTH	0.439	2.956	0.099
	Retraction	0.956	0.000	0.435

^a^*h*–haplotype diversity; *π*—nucleotide diversity;

^b^ PLAH, Pleistocene Arc Hypothesis; PPPH, Amazonian SDF Hypothesis; Both, pattern matching both hypotheses PLAH+PPPH; Retraction, pattern matching a strong range retraction. See Fig 2 for details about the demographic scenarios;

^c^(P) two-tailed probability;

^d^Δ*AIC* Akaike Information Criterion delta;

^e^ *AICw* Akaike Information Criterion weights.

### Spatial patterns in genetic diversity

Differentiation among populations was not significantly correlated with geographic distance for both chloroplast DNA (Mantel Test, r^2^ = 0.0172, p = 0.175) and ITS (r^2^ = 0.0037, p = 0.516).

**Fig 5:**
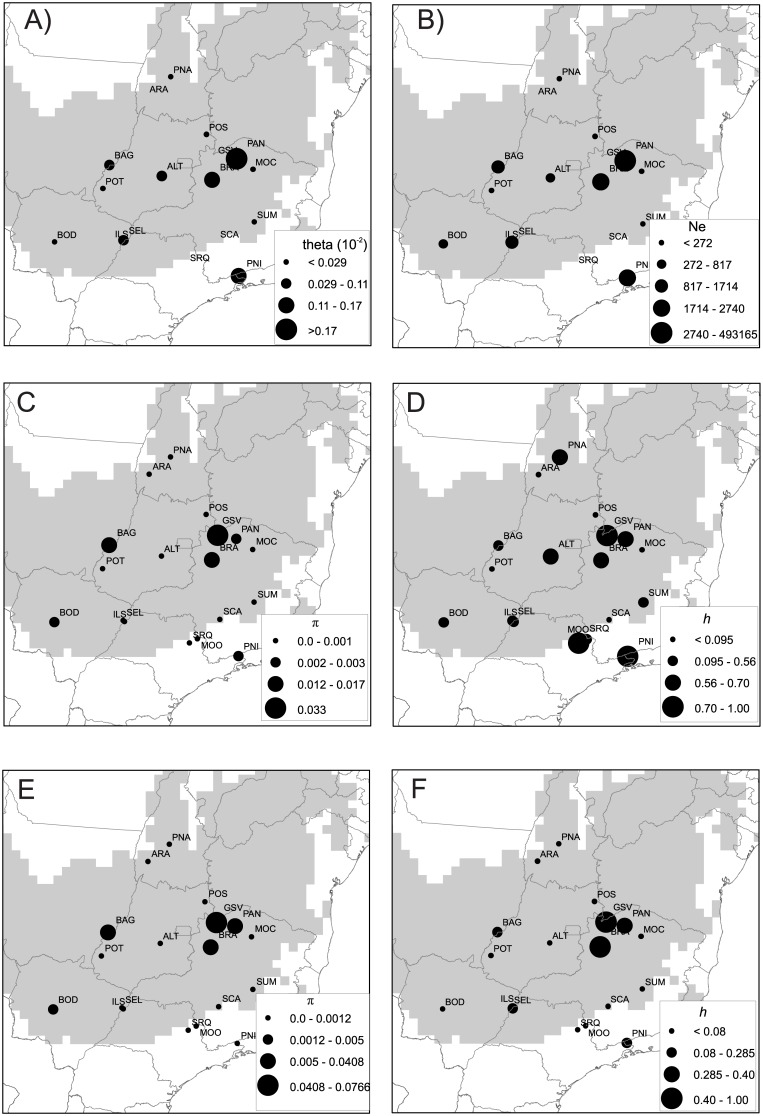
Spatial distribution of genetic diversity for *Tabebuia roseoalba* in relation to the historical refugium, i.e. areas climatically suitable throughout the time (in gray), based on the sequence of 235 individuals from 18 populations. (**A**) mutation parameter theta (*θ*). (**B**) Effective population size (*N*_*e*_). (**C**) Nucleotide diversity (*π*) for cpDNA. (**D**) Haplotype diversity (*h*) for cpDNA. (**E**) Nucleotide diversity (*π*) for ITS. (**D**) Haplotype diversity (*h*) for ITS. Circumference sizes are proportional to the value of genetic parameter, following the figure legends.

Quantile regressions showed clear effects of climate change on the genetic diversity of *T*. *roseoalba*, especially for ITS genome. Populations in more climatically suitable areas during the LGM, mid-Holocene and present-day showed higher *θ* (theta, mutation parameter), effective population size (*N*_*e*_) and haplotype (*h*) and nucleotide (π) diversities, especially for higher quantiles (Fig 5; see also S5 and S6 Figs). However, *θ* and *N*_*e*_ showed negative relationships with suitability at 21 ka (S5 Fig). Haplotype (*h*) diversity for chloroplast genome was not related with suitability.

Likewise, *θ*, *N*_*e*_, *h* and π for ITS decreased with distances from the centroid of the refugium and higher values are associated with populations in more stable areas, where climatic suitability have been unchanged since the LGM (S7 and S8 Figs).

## Discussion

### Testing the dry forest refugia hypothesis

Our findings from phylogeographic analyses of *T*. *roseoalba* do not support the hypotheses of modern-day dry forest refugia in South America, neither under its original ideas (Pleistocene Arc Hypothesis—PLAH or Amazonian SDF Hypothesis–PPPH) nor under its alternative arrangement combining both dynamics (PLAH + PPPH). Evidence from coalescent simulations and paleodistribution modeling indicate, actually, that the phylogeographic pattern of *T*. *roseoalba* is the result of a smaller geographic range at the LGM in comparison with present-day. Contrary to the originally expected by dry forest refugia hypothesis, the low haplotype and nucleotide diversity currently found in many populations of *T*. *roseoalba* is consistent with a demographic scenario of range retraction instead of a widely and continuously distributed SDTF during the LGM.

However, a range shift likewise the ‘Amazonian SDF Hypothesis’ (PPPH) may not be definitively disregarded. Firstly, because *T*. *roseoalba* occurs only on areas of high fertile soil, such as calcareous rocks, its range shift might have been less pronounced than other SDTF species such as *T*. *impetiginosa* [9]. Moreover, the coarse spatial resolution of predictors, like layers from paleoclimatic simulations and soil fertility estimates, preclude us of deeply evaluate the influences of climate change and soil parameters on species distribution at local scales by using ENMs. Thus, a range shift of *T*. *roseoalba* toward the Amazon Basin may have been favored at local scales, being weakly or not captured in our modeling approach. Indeed, quantile regressions showed a clinal variation in the genetic diversity of *T*. *roseoalba* for ITS, as expected for range shift. Range shift may lead to spatial genome assortment due to lead edge colonization, leading to loss and gradients in genetic diversity [60]. The spread of low-frequency allele during range shift may also cause low genetic diversity [61,62].

Secondarily, according to Pennington *et al*. [6], the Amazonian SDTF hypothesis (PPPH) is based on the distribution of some typical species from SDTFs (and not all SDTF species) that occur nowadays in low frequencies in areas of more fertile soils throughout the Amazon basin, at the local scales. Dynamics of water rivers and sea level during the Pleistocene glaciations would have exposed areas for the colonization by SDTF species, favoring the dispersal of such specific species into Amazon basin [6,63]. Thus, *T*. *roseoalba* might still be a SDTF species not universally favored by the dynamics of rivers throughout the Amazon basin. Anyway, our findings do not refute the Amazonian SDTF hypothesis (PPPH) at all, but do not support it as the most likely scenario for *T*. *roseoalba* (see similar results in [18 and 64]). Ultimately, both PPPH and range retraction hypotheses predict smaller populations at the LGM than in present-day, differing by range shift from PPPH. We conclude, therefore, that population contraction must be the general scenario explaining the demographic dynamics of *T*. *roseoalba* during the glacial phases, regardless of spatial shifts of SDTFs through the time.

Contrary to our results for *T*. *roseoalba*, the phylogeography of other SDTF species such as *Astronium urundeuva* [13] and *T*. *impetiginosa* [9] show clear evidence of range expansion during Pleistocene glacial times and suggest that the present distribution is a climatic relict of an ancient widely distributed population, supporting thus the modern-day dry forest refugia hypothesis. Contrasting results from other studies suggest that the response of SDTFs to the Quaternary climate changes was highly complex and may differ among SDTF plant [14,17] and animal [64] species. Actually, species-specific responses to Quaternary climate change have been longer proposed for other biomes in South America [65], and the own phylogeny and paleoecological data of SDTF species show both vicariance and long-distance dispersal as responsible for diversification in SDTFs in South America for both plants [7,8,18,66] and animals [64]. Indeed, the retraction of SDTF species during the LGM may have imposed restrictions to gene flow not only for plant species, as shown here, but also to animal species (e.g., [64]). In this study, we found no evidence of recent dispersal among *T*. *roseoalba* populations, although ancient dispersal may have happened before population retraction at the Pre-Illinoian (see next section below).

SDTFs are one of the most threaten ecosystems in the world [10]. Although they originally occupied ~ 42% of the tropical and subtropical forest regions, currently most of their remaining areas are in South America (~ 54.2%), mainly in northeast and central Brazil and in southeast Bolivia, Paraguay and northern Argentina [1]. In Brazil, most remaining areas of SDTFs are threatened mainly by agricultural expansion, harvesting for wood products and the increase of fire frequency due to agricultural practices [11]. The response of *T*. *roseoalba* to Quaternary climate changes showed in the present work may evince a potential constrain to respond to future climate changes. Large effects of climate changes on geographical ranges and shift of SDTF' species are predicted elsewhere [11]. Because *T*. *roseoalba* occurs only in highly fertile soils, this may hold back population migration and persistence threaten its long-term conservation.

### Spatial genetic structure

Populations in more climatically suitable areas during the LGM, mid-Holocene and present-day presented higher genetic diversity suggesting that stable areas were important refugia for *T*. *rosealba*. However, populations in more instable areas in southeastern and northwestern edges of the historical refugium (PNI, MOO, SQR, PNA) had high genetic diversity for chloroplast genome. We hypothesize that these populations at the edge of historical refugium may represent ‘stable rear edges’, i.e. relict populations that have persisted in suitable local habitats across the Quaternary glaciation cycles [67,68]. Differences in genetic diversity between chloroplast and ITS are most likely due to evolution rates and concerted evolution in ITS. Despite differences in effective population size and inheritance, chloroplast genome showed higher diversity than ITS nrDNA, which has four times the effective size of chloroplast genome and higher mutation rates. This may also be due to concerted evolution in nrDNA that may homogenize copies decreasing genetic variation [69].

Results on EBSP analysis and Fu’s neutrality test also showed demographic population retraction followed by expansion. EBSP showed that a demographic retraction started at ~ 588 ka (see Fig 3a), at the Middle Pleistocene, in the Pre-Illinoian Stage (the Cromerian Complex ~866 kyr BP to 478 kyr BP) [70], a sequence of three glacial periods followed by the most severe glaciation of the Pleistocene, the Anglian Stage (pre-Illinoian B) that lasted from ~478 ka to 424 ka [70]. The Anglian glaciation might have greater effects on species distribution than the more recent glacial period, the Wisconsin Glaciation (~ 110 ka to 12 ka). We hypothesize that range distribution of *T*. *roseoalba* was wider and started to shrink at the Pre-Illinoian glaciations due to the colder and drier conditions attaining its lower range and effective population size at the LGM. With the warmer and moisture conditions after the LGM, *T*. *rosealba* expanded its range, without secondary contact, evinced by the lack of gene flow. Populations of *T*. *roseoalba* were highly differentiated and both phylogenetic relationship among haplotypes and coalescent tree showed incomplete lineage sorting with populations geographically distant sharing a common ancestral. This result suggest that population of *T*. *roseoalba* were more connected before the Pre-Illinoian. Thus, the negligible gene flow among all population pairs (less than 1.0 migrant per generation) also indicates that the incongruence between the phylogenetic clades and geography may be the consequence of incomplete lineage sorting due to sundering [71]. In accordance, the lack of correlation between genetic differentiation and Bayesian clustering and geographical distance are evidences of both incomplete lineage sorting and negligible gene flow. The high migration into population PAN is most likely due to the large effective population size leading to high effect of migration, despite low migration rates. We believe that our results are not an artifact of statistical sampling because populations with higher sample size, such as BOD, ILS, POS and POT had low number of haplotypes for both cpDNA and ITS. Indeed, these populations showed significant signal of population size retraction.

Our results also showed an ancient origin of *T*. *roseoalba* lineages, dated from ~ 4.9 Ma. Different from *T*. *impetiginosa*, which lineages from the Southeast Brazil (SUM) first diverged at ~4.9 Myr BP [9], *T*. *roseoalba* lineages from central east Brazil (BRA and GSV) diverged first. Major divergences at geographical scale occurred in the Lower Pleistocene (Calabrian Stage, 1.8 Myr BP to 781 kyr BP) [70], but at regional and local scale, major divergences occurred in the Middle Pleistocene, coinciding with population size reduction showed by EBSP. Thus, the range retraction during the LGM, indicated by the paleodistribution modeling and by the simulation of demographic scenarios, matches the regional process of differentiation indicated by the coalescent tree with no later secondary contact.

## Concluding Remarks

Our results based on coalescent simulation and ecological niche modeling strongly support a retraction in geographical range and effective population size of *T*. *roseoalba* during the LGM, contrary to the expected by modern-day dry forest refugia hypothesis. Contrasting results for related SDTF species, like the congeneric *T*. *roseoalba* and *T*. *impetiginosa*, indicate that the effects of Quaternary climate changes on distribution dynamics of SDTF in South America is more complex than previously expected. Our study shows that SDTF responses to Quaternary climate changes should be species-specifically investigated, instead of considering multiple species in association or entire biomes. Finally, we showed here that phylogeographic analyses coupled with ecological niche modeling and coalescent simulations can be a very powerful framework for evaluating alternative hypotheses from individualistic species and potentially useful for disentangling mechanisms involved in the origin of the disjunct distribution of SDTFs.

## Supporting Information

S1 Fig Geographical distribution of *Tabebuia roseoalba* based on the occurrence records from GBIF (Global Biodiversity Information Facility http://www.gbif.org/). Map adapted from Collevatti et al. (2012).
(DOCX)

S2 Fig Phylogenetic relationships among haplotypes for (a) ITS and (b) cpDNA, using median-joining network. (DOCX)

S3 Fig Average and 0.95 confidence interval among the 60 maps of (a) range size and (b) shift (difference of range size among time periods in number of cells) predicted for *Tabebuia roseoalba* at LGM (21 ka), mid-Holocene (6 ka), and present-day (0 ka). (DOCX)

S4 Fig Maps of uncertainty (relative sum of squares) for the modeling components of *Tabebuia roseoalba*, (A) Time, (B) Atmosphere-Ocean Global Circulation Models (AOGCMs), (C) Ecological Niche Models (ENMs), (D) interaction of AOGCM and ENM. (DOCX)

S5 Fig Quantile regression between effective population size (*N*_*e*_) and mutation parameter theta (*θ*) for ITS sequences and climate suitability for 18 populations of *Tabebuia roseoalba* for present-day (0 k), mid-Holocene (6 k) and Last Glacial Maximum (21 k). (DOCX)

S6 Fig Quantile regression between haplotype (*h*) and nucleotide (*π*) diversities for ITS sequences and climate suitability for 18 populations of *Tabebuia roseoalba* for present-day (0 k), mid-Holocene (6 k) and Last Glacial Maximum (21 k). (DOCX)

S7 Fig Quantile regression between effective population size (*N*_*e*_) and mutation parameter theta (*θ*) for ITS sequences and distance to the centroid of the historical refugium and climate stability, for 18 populations of *Tabebuia roseoalba*. (DOCX)

S8 Fig Quantile regression between haplotype (*h*) and nucleotide (*π*) diversities for ITS sequences and distance to the centroid of the historical refugium and climate stability, for 18 populations of *Tabebuia roseoalba*. (DOCX)

S1 Table Sampling localities of *Tabebuia roseoalba* populations and outgroups used for phylogeographic analyzes. (DOCX)

S2 Table Contemporary occurrence records (33) of *Tabebuia roseoalba* represented by the centroid of grid cells across the Neotropics used in the ecological niche modelling (ENM). (DOCX)

S3 Table Details on the paleoclimatic simulations (AOGCMs) used in the ecological niche modeling of *Tabebuia roseoalba*. (DOCX)

S4 Table Ecological niche modeling methods used to estimate *Tabebuia roseoalba* potential distribution. (DOCX)

S5 Table Values of True Skill Statistics (TSS) with mean and confidence intervals (CI) for all ENM x AOGCM’s combinations from ecological niche modeling of *Tabebuia roseoalba*. (DOCX)

S6 Table Pairwise *F*_*ST*_ among the 18 populations of *Tabebuia roseoalba* sampled in Brazil. Chloroplast *F*_*ST*_ below diagonal and nuclear ITS above diagonal.
(DOCX)

S7 Table Number of migrants per generation (*N*_*e*_*m*) for the 13 populations of *Tabebuia roseoalba* in Brazil, based on Bayesian coalescent analysis. Migration direction is from populations in the columns into populations in the rows.
(DOCX)

S8 Table Credibility interval (95%) of the number of migrants per generation (see S7 Table for the number of migrants) for the 18 populations of *Tabebuia roseoalba* in Brazil, based on Bayesian coalescent analysis. (DOCX)

S9 Table Uncertainty of modeling components on ENM predictions for *Tabebuia roseoalba* as revealed by hierarchical ANOVA. (DOCX)
