# Supplementary material for: Coalescent Simulation and Paleodistribution Modeling for Tabebuia rosealba Do Not Support South American Dry Forest Refugia Hypothesis
Source: PLoS One. 2016 Jul 26;11(7):e0159314. doi: 10.1371/journal.pone.0159314 (PMC4961443; doi:10.1371/journal.pone.0159314)
Supplement: S1 Fig — Map adapted from Collevatti et al. (2012). (DOCX) [file pone.0159314.s001.docx]

**Coalescent simulation and paleodistribution modeling for *Tabebuia rosealba* do not support South American dry forest refugia hypothesis**

Warita Alves de Melo^1^, Matheus S. Lima-Ribeiro^2^, Levi Carina Terribile^2^, Rosane G. Collevatti^1*^


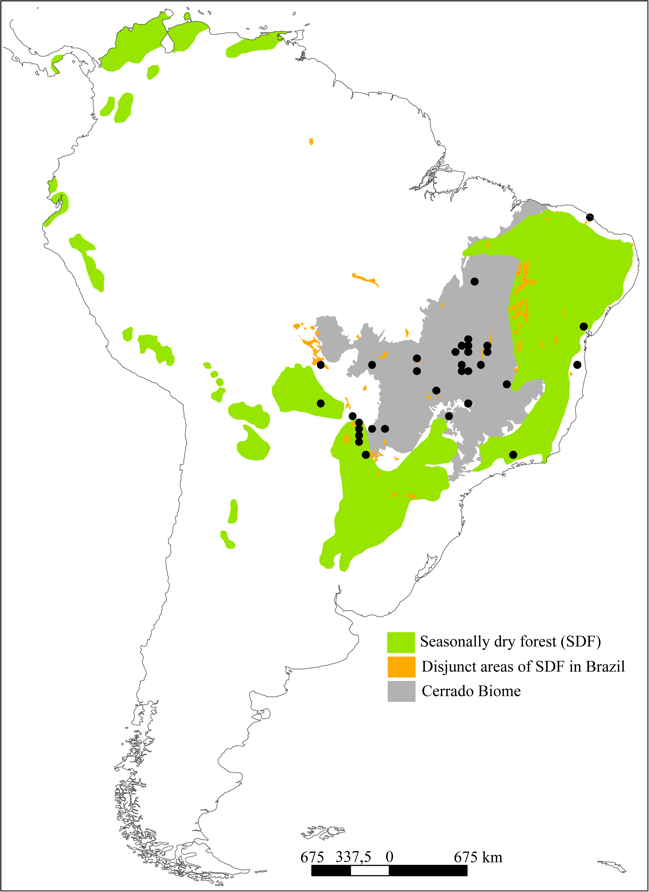


**S1 Fig**. Geographical distribution of *Tabebuia roseoalba* based on the occurrence records from GBIF (Global Biodiversity Information Facility <http://www.gbif.org/>). Map adapted from Collevatti et al. (2012).
