# Supplementary material for: Coalescent Simulation and Paleodistribution Modeling for Tabebuia rosealba Do Not Support South American Dry Forest Refugia Hypothesis
Source: PLoS One. 2016 Jul 26;11(7):e0159314. doi: 10.1371/journal.pone.0159314 (PMC4961443; doi:10.1371/journal.pone.0159314)
Supplement: S2 Fig — (DOCX) [file pone.0159314.s002.docx]

**Coalescent simulation and paleodistribution modeling for *Tabebuia rosealba* do not support South American dry forest refugia hypothesis**

Warita Alves de Melo^1^, Matheus S. Lima-Ribeiro^2^, Levi Carina Terribile^2^, Rosane G. Collevatti^1*^

(a)


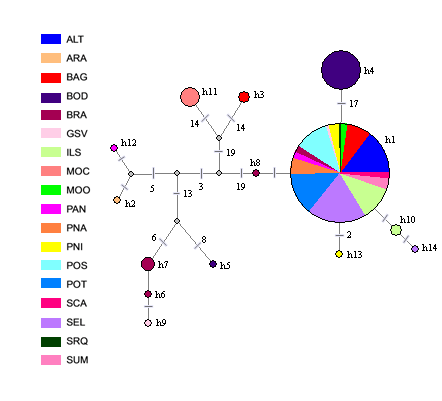


(b)


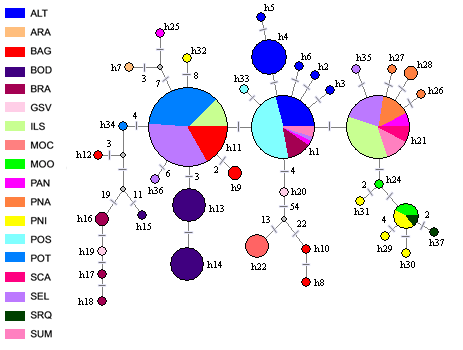


**S2 Fig.** Phylogenetic relationships among haplotypes for (**a**) ITS and (**b**) cpDNA, using median-joining network. Circumference size is proportional to the haplotype frequency. Number of mutations is shown along lines in the network; small black circles are the median vectors. Different colors were assigned for each population according to the figure legend.
