# Supplementary material for: Coalescent Simulation and Paleodistribution Modeling for Tabebuia rosealba Do Not Support South American Dry Forest Refugia Hypothesis
Source: PLoS One. 2016 Jul 26;11(7):e0159314. doi: 10.1371/journal.pone.0159314 (PMC4961443; doi:10.1371/journal.pone.0159314)
Supplement: S3 Fig — (DOCX) [file pone.0159314.s003.docx]

**Coalescent simulation and paleodistribution modeling for *Tabebuia rosealba* do not support South American dry forest refugia hypothesis**

Warita Alves de Melo^1^, Matheus S. Lima-Ribeiro^2^, Levi Carina Terribile^2^, Rosane G. Collevatti^1*^


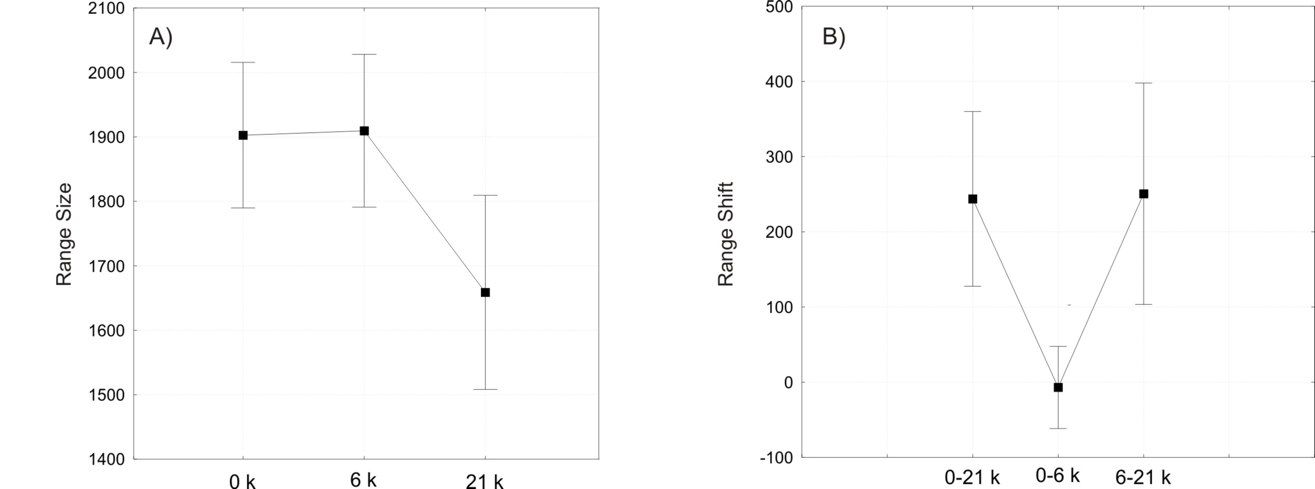


**S3 Fig.** Average and 0.95 confidence interval among the 60 maps of **(a)** range size and **(b)** shift (difference of range size among time periods in number of cells) predicted for *Tabebuia roseoalba* at LGM (21 ka), mid-Holocene (6 ka), and present-day (0 ka). Note that a general scenario of range expansion though time (positive range shifts) is supported.
