# Supplementary material for: Coalescent Simulation and Paleodistribution Modeling for Tabebuia rosealba Do Not Support South American Dry Forest Refugia Hypothesis
Source: PLoS One. 2016 Jul 26;11(7):e0159314. doi: 10.1371/journal.pone.0159314 (PMC4961443; doi:10.1371/journal.pone.0159314)
Supplement: S4 Fig — (DOCX) [file pone.0159314.s004.docx]

**Coalescent simulation and paleodistribution modeling for *Tabebuia rosealba* do not support South American dry forest refugia hypothesis**

Warita Alves de Melo^1^, Matheus S. Lima-Ribeiro^2^, Levi Carina Terribile^2^, Rosane G. Collevatti^1*^


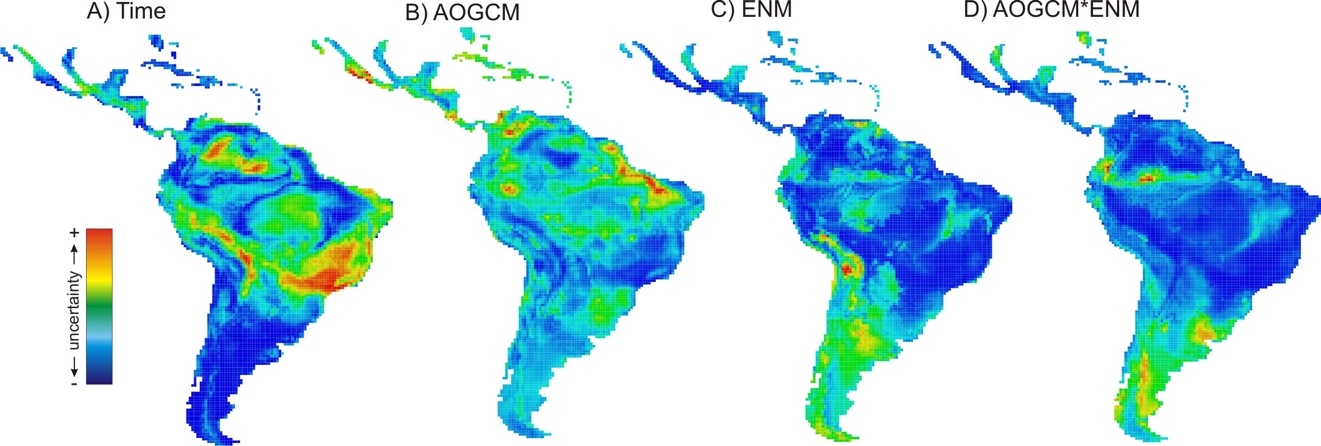


**S4 Fig.** Maps of uncertainty (relative sum of squares) for the modeling components of *Tabebuia roseoalba*, **(A)** Time, **(B)** Atmosphere-Ocean Global Circulation Models (AOGCMs), **(C)** Ecological Niche Models (ENMs), (**D**) interaction of AOGCM and ENM.
