# Supplementary material for: Coalescent Simulation and Paleodistribution Modeling for Tabebuia rosealba Do Not Support South American Dry Forest Refugia Hypothesis
Source: PLoS One. 2016 Jul 26;11(7):e0159314. doi: 10.1371/journal.pone.0159314 (PMC4961443; doi:10.1371/journal.pone.0159314)
Supplement: S7 Fig — (DOCX) [file pone.0159314.s007.docx]

**Coalescent simulation and paleodistribution modeling for *Tabebuia rosealba* do not support South American dry forest refugia hypothesis**

Warita Alves de Melo^1^, Matheus S. Lima-Ribeiro^2^, Levi Carina Terribile^2^, Rosane G. Collevatti^1*^


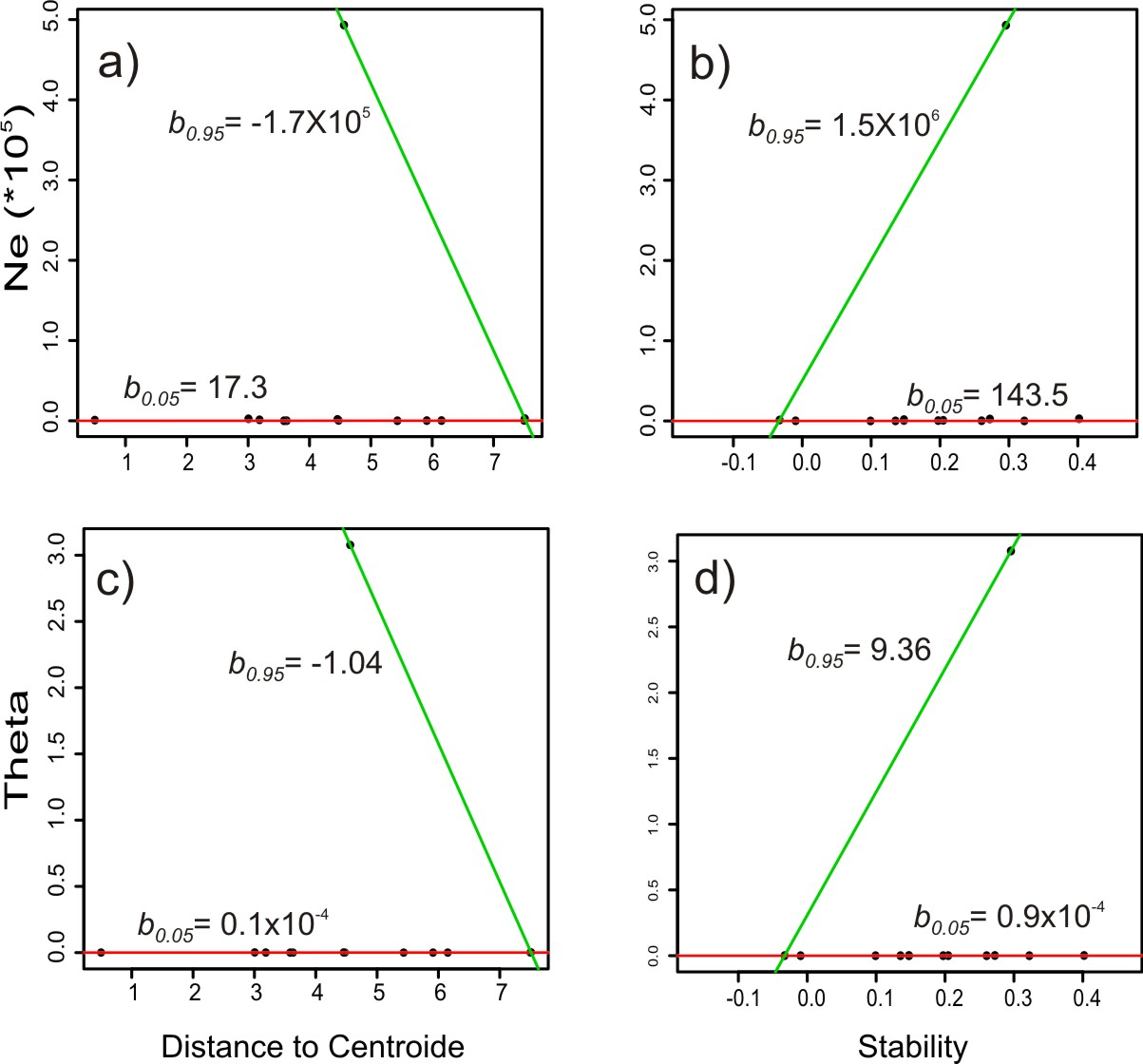


**S7 Fig.** Quantile regression between effective population size (*N_e_*) and mutation parameter theta (*θ*) for ITS sequences and distance to the centroid of the historical refugium and climate stability, for 18 populations of *Tabebuia roseoalba.* Green line shows the fitness for 95% (upper) and red line for 10% (down) quantiles.
