# Supplementary material for: Coalescent Simulation and Paleodistribution Modeling for Tabebuia rosealba Do Not Support South American Dry Forest Refugia Hypothesis
Source: PLoS One. 2016 Jul 26;11(7):e0159314. doi: 10.1371/journal.pone.0159314 (PMC4961443; doi:10.1371/journal.pone.0159314)
Supplement: S1 Table — (DOCX) [file pone.0159314.s009.docx]

**Coalescent simulation and paleodistribution modeling for *Tabebuia rosealba* do not support South American dry forest refugia hypothesis**

Warita Alves de Melo^1^, Matheus S. Lima-Ribeiro^2^, Levi Carina Terribile^2^, Rosane G. Collevatti^1*^

**S1 Table.** Sampling localities of *Tabebuia roseoalba* populations and outgroups used for phylogeographic analyzes. N, number of individuals sampled.

| **Code** | | **Locality** | | **N** | | **Longitude Latitude** | |  |  |
| --- | --- | --- | --- | --- | --- | --- | --- | --- | --- |
| ALT | | Altamiro Pacheco/GO | | 29 | | -16.5389 | | -49.1382 | |
| ARA | | Fazenda Araguaia/TO | | 1 | | -11.6720 | | -49.8744 | |
| BAG | | Barra do Garças/MT | | 17 | | -15.8900 | | -52.2569 | |
| BOD | | Serra da Bodoquena/MS | | 25 | | -20.4762 | | -55.5208 | |
| BRA | | Brasilândia/MG | | 9 | | -16.7649 | | -46.1416 | |
| GSV | | Grande Sertão Veredas/MG | | 2 | | -15.3071 | | -45.8033 | |
| ILS | | Ilha Solteira/RJ | | 23 | | -20.4328 | | -51.3428 | |
| MOC | | Montes Claros/MG | | 6 | | -16.1492 | | -43.7191 | |
| MOO | | Mococa/SP | | 4 | | -21.4678 | | -47.0050 | |
| PAN | | Pandeiros/MG | | 5 | | -15.5149 | | -44.6924 | |
| PNA | | Porto Nacional/TO | | 10 | | -10.6516 | | -48.5983 | |
| PNI | | Parque Nacional do Itatiaia/SP | | 7 | | -22.4914 | | -44.5592 | |
| POS | | Posse/GO | | 21 | | -14.0634 | | -46.4860 | |
| POT | | Portelândia/GO | | 26 | | -17.2958 | | -52.6419 | |
| SEL | | Selvilha/SP | | 38 | | -20.3669 | | -51.4189 | |
| SCA | | Pains/MG | | 3 | | -20.3053 | | -45.6753 | |
| SRQ | | Santa Rita do Passa Quatro/SP | | 2 | | -21.7100 | | -47.4778 | |
| SUM | | Sumidouro/MG | | 7 | | -19.2842 | | -43.6328 | |
| CAN | | *Cybistax antisyphilitica* (Sapucaia/RJ) | | 1 | | -21.995 | | -42.9138 | |
| HIM | | *Handroanthus impetiginosus* (Sumidouro/MG) | | 1 | | -19.2842 | | -43.6328 | |
| TOC | | *Tabebuia ochraceae* (Sumidouro/MG) | | 1 | | -19.2842 | | -43.6328 | |
