# Supplementary material for: Coalescent Simulation and Paleodistribution Modeling for Tabebuia rosealba Do Not Support South American Dry Forest Refugia Hypothesis
Source: PLoS One. 2016 Jul 26;11(7):e0159314. doi: 10.1371/journal.pone.0159314 (PMC4961443; doi:10.1371/journal.pone.0159314)
Supplement: S2 Table — (DOCX) [file pone.0159314.s010.docx]

**Coalescent simulation and paleodistribution modeling for *Tabebuia rosealba* do not support South American dry forest refugia hypothesis**

Warita Alves de Melo^1^, Matheus S. Lima-Ribeiro^2^, Levi Carina Terribile^2^, Rosane G. Collevatti^1*^

**S2 Table.** Contemporary occurrence records (33) of *Tabebuia roseoalba* represented by the centroid of grid cells across the Neotropics used in the ecological niche modelling (ENM)**.**

| **Longitude** | **Latitude** |
| --- | --- |
| -59.6 | -18.3 |
| -59.6 | -15.3 |
| -57.1 | -19.3 |
| -56.6 | -21.3 |
| -56.6 | -20.8 |
| -56.6 | -20.3 |
| -56.6 | -19.8 |
| -56.1 | -22.3 |
| -55.6 | -20.3 |
| -55.6 | -15.3 |
| -54.6 | -20.3 |
| -52.1 | -15.8 |
| -52.1 | -14.8 |
| -50.6 | -17.3 |
| -49.6 | -19.3 |
| -49.1 | -14.3 |
| -48.6 | -15.8 |
| -48.6 | -15.3 |
| -48.6 | -13.8 |
| -48.1 | -18.3 |
| -48.1 | -15.8 |
| -48.1 | -14.3 |
| -48.1 | -13.8 |
| -48.1 | -13.3 |
| -47.6 | -8.8 |
| -47.1 | -15.3 |
| -46.6 | -14.3 |
| -46.6 | -13.8 |
| -45.1 | -16.8 |
| -44.6 | -22.3 |
| -39.6 | -15.3 |
| -39.1 | -12.3 |
| -38.6 | -3.8 |
