# Supplementary material for: Coalescent Simulation and Paleodistribution Modeling for Tabebuia rosealba Do Not Support South American Dry Forest Refugia Hypothesis
Source: PLoS One. 2016 Jul 26;11(7):e0159314. doi: 10.1371/journal.pone.0159314 (PMC4961443; doi:10.1371/journal.pone.0159314)
Supplement: S3 Table — (DOCX) [file pone.0159314.s011.docx]

**Coalescent simulation and paleodistribution modeling for *Tabebuia rosealba* do not support South American dry forest refugia hypothesis**

Warita Alves de Melo^1^, Matheus S. Lima-Ribeiro^2^, Levi Carina Terribile^2^, Rosane G. Collevatti^1*^

**S3 Table.** Details on the paleoclimatic simulations (AOGCMs) used in the ecological niche modeling of *Tabebuia roseoalba.*

| **Model ID** | **Modeling Center** | **Resolution*** | **Source** | **Year** |
| --- | --- | --- | --- | --- |
| CCSM4 | University of Miami – RSMAS. USA | 0.9° × 1.25° | CMIP5/PMIP3 | 2012 |
| CNRM-CM5 | Centre National de Recherches Meteorologiques / Centre Europeen de Recherche et Formation Avancees en Calcul Scientifique. France | 1.4° x 1.4° | CMIP5/PMIP3 | 2012 |
| MIROC-ESM | Atmosphere and Ocean Research Institute (University of Tokyo). National Institute for Environmental Studies. and Japan Agency for Marine-Earth Science and Technology. Japan | 2.8° × 2.8° | CMIP5/PMIP3 | 2012 |
| MPI-ESM-P | Max Planck Institute for Meteorology, Germany | 1.9° x 1.9° | CMIP5/PMIP3 | 2011 |
| MRI-CGCM3 | Meteorological Research Institute. Japan | 1.1° x 1.1° | CMIP5/PMIP3 | 2012 |

* longitude × latitude

CMIP5 – Coupled Model Intercomparison Project. Phase 5 (<http://cmip-pcmdi.llnl.gov/>)

PMIP3 – Paleoclimate Modelling Intercomparison Project. Phase 3 (<http://pmip3.lsce.ipsl.fr/>
