# Supplementary material for: Coalescent Simulation and Paleodistribution Modeling for Tabebuia rosealba Do Not Support South American Dry Forest Refugia Hypothesis
Source: PLoS One. 2016 Jul 26;11(7):e0159314. doi: 10.1371/journal.pone.0159314 (PMC4961443; doi:10.1371/journal.pone.0159314)
Supplement: S4 Table — (DOCX) [file pone.0159314.s012.docx]

**Coalescent simulation and paleodistribution modeling for *Tabebuia rosealba* do not support South American dry forest refugia hypothesis**

Warita Alves de Melo^1^, Matheus S. Lima-Ribeiro^2^, Levi Carina Terribile^2^, Rosane G. Collevatti^1*^

**S4 Table.** Ecological niche modeling methods used to estimate *Tabebuia roseoalba* potential distribution.

| **Method** | **Species data type** |
| --- | --- |
| Bioclimatic Envelope (BIOCLIM) | Presence only |
| Ecological Niche Factor Analysis (ENFA) | Presence only |
| Euclidian Distance (EuclidDist) | Presence only |
| Generalized Linear Models (GLM) | Presence and absence |
| Gower Distance (GowerDist) | Presence only |
| Mahalanobis Distance (MahalDist) | Presence only |
| Maximum Entropy (Maxent) | Presence/background |
| Generalized additive models (GAM) | Presence and absence |
| Flexible discriminant analysis (FDA) | Presence and absence |
| Multivariate adaptive regression splines (MARS) | Presence and absence |
| Neural Networks (NNET) | Presence and absence |
| Random Forest (RNDFOR) | Presence and absence |
