# Supplementary material for: Coalescent Simulation and Paleodistribution Modeling for Tabebuia rosealba Do Not Support South American Dry Forest Refugia Hypothesis
Source: PLoS One. 2016 Jul 26;11(7):e0159314. doi: 10.1371/journal.pone.0159314 (PMC4961443; doi:10.1371/journal.pone.0159314)
Supplement: S5 Table — (DOCX) [file pone.0159314.s013.docx]

**Coalescent simulation and paleodistribution modeling for *Tabebuia rosealba* do not support South American dry forest refugia hypothesis**

Warita Alves de Melo^1^, Matheus S. Lima-Ribeiro^2^, Levi Carina Terribile^2^, Rosane G. Collevatti^1*^

**S5 Table.** Values of True Skill Statistics (TSS) with mean and confidence intervals (CI) for all ENM x AOGCM’s combinations from ecological niche modeling of *Tabebuia roseoalba*.

|  |  | **AOGCMs** | | | | | |
| --- | --- | --- | --- | --- | --- | --- | --- |
|  |  | **CCSM** | **CNRM** | **MIROC** | **MPI** | **MRI** | ***CI*** |
| **ENM Algorithms** | **BioClim** | 0.5212 | 0.5117 | 0.399 | 0.6293 | 0.4795 | *0.43 - 0.58* |
|  | **ENFA** | 0.3562 | 0.4153 | 0.276 | 0.4178 | 0.3458 | *0.31 - 0.41* |
|  | **EuclidDist** | 0.4682 | 0.3985 | 0.3218 | 0.5902 | 0.5165 | *0.37 - 0.55* |
|  | **FDA** | 0.519 | 0.5157 | 0.4967 | 0.6117 | 0.513 | *0.49 - 0.57* |
|  | **GAM** | 0.5955 | 0.6115 | 0.5532 | 0.6402 | 0.593 | *0.57 - 0.63* |
|  | **GLM** | 0.6247 | 0.5312 | 0.5545 | 0.622 | 0.536 | *0.53 - 0.61* |
|  | **GowerDist** | 0.4902 | 0.4775 | 0.2713 | 0.6482 | 0.5142 | *0.36 - 0.60* |
|  | **MahalanobisDist** | 0.4213 | 0.4368 | 0.2905 | 0.6053 | 0.5137 | *0.35 - 0.55* |
|  | MARS | 0.5403 | 0.5085 | 0.4587 | 0.589 | 0.4983 | *0.48 - 0.56* |
|  | MaxEnt | 0.5043 | 0.613 | 0.459 | 0.6777 | 0.5498 | *0.48 - 0.64* |
|  | NNet | 0.457 | 0.5202 | 0.3718 | 0.5348 | 0.4612 | *0.41 - 0.53* |
|  | RndFor | 0.6187 | 0.5802 | 0.5867 | 0.6368 | 0.5845 | *0.58 - 0.62* |
|  | **CI** | 0.46 - 0.55 | 0.47 - 0.55 | 0.35 - 0.48 | 0.56 - 0.64 | 0.47 - 0.54 |  |
