# Supplementary material for: Coalescent Simulation and Paleodistribution Modeling for Tabebuia rosealba Do Not Support South American Dry Forest Refugia Hypothesis
Source: PLoS One. 2016 Jul 26;11(7):e0159314. doi: 10.1371/journal.pone.0159314 (PMC4961443; doi:10.1371/journal.pone.0159314)
Supplement: S6 Table — Chloroplast FST below diagonal and nuclear ITS above diagonal. (DOCX) [file pone.0159314.s014.docx]

**Coalescent simulation and paleodistribution modeling for *Tabebuia rosealba* do not support South American dry forest refugia hypothesis**

Warita Alves de Melo^1^, Matheus S. Lima-Ribeiro^2^, Levi Carina Terribile^2^, Rosane G. Collevatti^1*^

**S6 Table.** Pairwise *F_ST_* among the 18 populations of *Tabebuia roseoalba* sampled in Brazil. Chloroplast *F_ST_* below diagonal and nuclear ITS above diagonal. Values followed by ns are not significant, *p* > 0.05.

|  | **ALT** | **BAG** | **BOD** | **BRA** | **GSV** | **ILS** | **MOC** | **MOO** | **PAN** | **PNA** | **PNI** | **POS** | **POT** | **SCA** | **SEL** | **SRQ** | **SUM** |
| --- | --- | --- | --- | --- | --- | --- | --- | --- | --- | --- | --- | --- | --- | --- | --- | --- | --- |
| **ALT** | 0.000 | 0.227 | 0.655 | 0.579 | 0.835 | 0.452 | 0.991 | 0.779 | 0.401 | 0.614 | 0.613 | 0.304 | 0.729 | 0.598 | 0.436 | 0.808 | 0.481 |
| **BAG** | 0.227 | 0.000 | 0.272 | 0.222 | 0.207 | 0.155 | 0.803 | 0.135 | 0.026 ^ns^ | 0.163 | 0.159 | 0.178 | 0.105 | 0.002 | 0.146 | 0.050 | 0.093 |
| **BOD** | 0.655 | 0.272 | 0.000 | 0.528 | 0.701 | 0.596 | 0.957 | 0.636 | 0.497 | 0.618 | 0.580 | 0.647 | 0.606 | 0.551 | 0.583 | 0.632 | 0.572 |
| **BRA** | 0.579 | 0.222 | 0.528 | 0.000 | 0.351 | 0.529 | 0.777 | 0.290 | 0.251 | 0.408 | 0.321 | 0.529 | 0.575 | 0.205 | 0.605 | 0.173 | 0.335 |
| **GSV** | 0.835 | 0.207 | 0.701 | 0.351 | 0.000 | 0.797 | 0.864 | 0.446 | 0.387 | 0.685 | 0.470 | 0.833 | 0.862 | 0.297 | 0.832 | 0.103 | 0.595 |
| **ILS** | 0.452 | 0.155 | 0.596 | 0.529 | 0.797 | 0.000 | 0.989 | 0.649 | 0.105 | 0.245 | 0.457 | 0.469 | 0.587 | 0.128 | 0.145 | 0.708 | 0.085 |
| **MOC** | 0.991 | 0.803 | 0.957 | 0.777 | 0.864 | 0.989 | 0.000 | 0.997 | 0.974 | 0.993 | 0.961 | 0.999 | 0.999 | 1.000 | 0.985 | 0.995 | 0.996 |
| **MOO** | 0.779 | 0.135 | 0.636 | 0.290 | 0.446 | 0.649 | 0.997 | 0.000 | 0.364 | 0.685 | 0.069 | 0.945 | 0.967 | 0.831 | 0.684 | 0.211 | 0.762 |
| **PAN** | 0.401 | 0.026 ^ns^ | 0.497 | 0.251 | 0.387 | 0.105 | 0.974 | 0.364 | 0.000 | 0.145 | 0.201 | 0.445 | 0.658 | 0.088 | 0.265 | 0.312 | 0.018 ^ns^ |
| **PNA** | 0.614 | 0.163 | 0.618 | 0.408 | 0.685 | 0.245 | 0.993 | 0.685 | 0.145 | 0.000 | 0.361 | 0.776 | 0.888 | 0.136 | 0.487 | 0.723 | 0.090 |
| **PNI** | 0.613 | 0.159 | 0.580 | 0.321 | 0.470 | 0.457 | 0.961 | 0.069 ^ns^ | 0.201 | 0.361 | 0.000 | 0.639 | 0.736 | 0.160 | 0.555 | 0.112 | 0.309 |
| **POS** | 0.304 | 0.178 | 0.647 | 0.529 | 0.833 | 0.469 | 0.999 | 0.945 | 0.445 | 0.776 | 0.639 | 0.000 | 0.922 | 0.919 | 0.408 | 0.949 | 0.730 |
| **POT** | 0.729 | 0.105 | 0.606 | 0.575 | 0.862 | 0.587 | 0.999 | 0.967 | 0.658 | 0.888 | 0.736 | 0.922 | 0.000 | 0.965 | 0.156 | 0.967 | 0.907 |
| **SCA** | 0.598 | 0.002 ^ns^ | 0.551 | 0.205 | 0.297 | 0.128 | 1.000 | 0.831 | 0.088 | 0.136 | 0.160 | 0.919 | 0.965 | 0.000 | 0.431 | 0.769 | 0.010 |
| **SEL** | 0.436 | 0.146 | 0.583 | 0.605 | 0.832 | 0.145 | 0.985 | 0.684 | 0.265 | 0.487 | 0.555 | 0.408 | 0.156 | 0.431 | 0.000 | 0.725 | 0.364 |
| **SRQ** | 0.808 | 0.050^ns^ | 0.632 | 0.173 | 0.103 | 0.708 | 0.995 | 0.211 | 0.312 | 0.723 | 0.112 | 0.949 | 0.967 | 0.769 | 0.725 | 0.000 | 0.773 |
| **SUM** | 0.481 | 0.093 | 0.572 | 0.335 | 0.595 | 0.085 | 0.996 | 0.762 | 0.018 | 0.090 | 0.309 | 0.730 | 0.907 | 0.010 ^ns^ | 0.364 | 0.773 | 0.000 |
