# Supplementary material for: Coalescent Simulation and Paleodistribution Modeling for Tabebuia rosealba Do Not Support South American Dry Forest Refugia Hypothesis
Source: PLoS One. 2016 Jul 26;11(7):e0159314. doi: 10.1371/journal.pone.0159314 (PMC4961443; doi:10.1371/journal.pone.0159314)
Supplement: S7 Table — Migration direction is from populations in the columns into populations in the rows. (DOCX) [file pone.0159314.s015.docx]

**Coalescent simulation and paleodistribution modeling for *Tabebuia rosealba* do not support South American dry forest refugia hypothesis**

Warita Alves de Melo^1^, Matheus S. Lima-Ribeiro^2^, Levi Carina Terribile^2^, Rosane G. Collevatti^1*^

**S7 Table.** Number of migrants per generation (*N_e_m*) for the 13 populations of *Tabebuia roseoalba* in Brazil, based on Bayesian coalescent analysis. Migration direction is from populations in the columns into populations in the rows. Note that all values of *N_e_m* are < 1.00 except for population PAN.

|  | **ALT** | **BAG** | **BOD** | **BRA** | **ILS** | **MOC** | **PAN** | **PNA** | **PNI** | **POS** | **POT** | **SEL** | **SUM** |
| --- | --- | --- | --- | --- | --- | --- | --- | --- | --- | --- | --- | --- | --- |
| **ALT** |  | 0.0144 | 0.0249 | 0.0404 | 0.0051 | 0.0069 | 0.0324 | 0.0465 | 0.0049 | 0.0463 | 0.0259 | 0.0049 | 0.0045 |
| **BAG** | 0.0545 |  | 0.0545 | 0.0064 | 0.0657 | 0.0068 | 0.0688 | 0.0070 | 0.0115 | 0.0279 | 0.0638 | 0.0650 | 0.0635 |
| **BOD** | 0.0041 | 0.0225 |  | 0.0028 | 0.0024 | 0.0024 | 0.0257 | 0.0189 | 0.0045 | 0.0027 | 0.0026 | 0.0026 | 0.0026 |
| **BRA** | 0.0949 | 0.0145 | 0.0156 |  | 0.0166 | 0.0140 | 0.0513 | 0.0137 | 0.0312 | 0.1387 | 0.0700 | 0.1408 | 0.1189 |
| **ILS** | 0.0097 | 0.0027 | 0.0025 | 0.0260 |  | 0.0025 | 0.0027 | 0.0266 | 0.0025 | 0.0241 | 0.0263 | 0.0134 | 0.0264 |
| **MOC** | 0.0021 | 0.0006 | 0.0004 | 0.0004 | 0.0004 |  | 0.0034 | 0.0030 | 0.0004 | 0.0007 | 0.0008 | 0.0005 | 0.0004 |
| **PAN** | 26.2200 | 22.7153 | 19.0874 | 70.6877 | 272.6680 | 24.1046 |  | 266.9395 | 21.3616 | 28.8947 | 25.5803 | 26.6480 | 201.1599 |
| **PNA** | 0.0049 | 0.0033 | 0.0036 | 0.0012 | 0.0013 | 0.0040 | 0.0021 |  | 0.0037 | 0.0028 | 0.0101 | 0.0013 | 0.0072 |
| **PNI** | 0.1254 | 0.0693 | 0.0625 | 0.0157 | 0.1198 | 0.0168 | 0.1539 | 0.1428 |  | 0.1443 | 0.0373 | 0.0166 | 0.0169 |
| **POS** | 0.0058 | 0.0007 | 0.0007 | 0.0022 | 0.0007 | 0.0007 | 0.0006 | 0.0046 | 0.0007 |  | 0.0057 | 0.0006 | 0.0063 |
| **POT** | 0.0004 | 0.0017 | 0.0003 | 0.0005 | 0.0018 | 0.0005 | 0.0001 | 0.0012 | 0.0004 | 0.0015 |  | 0.0013 | 0.0005 |
| **SEL** | 0.0931 | 0.0900 | 0.0094 | 0.0117 | 0.0882 | 0.0097 | 0.0519 | 0.0956 | 0.0160 | 0.0883 | 0.0895 |  | 0.0971 |
| **SUM** | 0.0017 | 0.0122 | 0.0020 | 0.0030 | 0.0075 | 0.0041 | 0.0015 | 0.0149 | 0.0094 | 0.0128 | 0.0016 | 0.0056 |  |
