# Supplementary material for: Coalescent Simulation and Paleodistribution Modeling for Tabebuia rosealba Do Not Support South American Dry Forest Refugia Hypothesis
Source: PLoS One. 2016 Jul 26;11(7):e0159314. doi: 10.1371/journal.pone.0159314 (PMC4961443; doi:10.1371/journal.pone.0159314)
Supplement: S8 Table — (DOCX) [file pone.0159314.s016.docx]

**Coalescent simulation and paleodistribution modeling for *Tabebuia rosealba* do not support South American dry forest refugia hypothesis**

Warita Alves de Melo^1^, Matheus S. Lima-Ribeiro^2^, Levi Carina Terribile^2^, Rosane G. Collevatti^1*^

**S8 Table.** Credibility interval (95%) of the number of migrants per generation (see Table S7 for the number of migrants) for the 18 populations of *Tabebuia roseoalba* in Brazil, based on Bayesian coalescent analysis. Credibility interval for migration is from populations in the columns into populations in the rows. All values are > 0.000 (approximate decimal places).

|  | **ALT** | | **BAG** | | **BOD** | | **BRA** | | **ILS** | | **MOC** | | **PAN** | | **PNA** | | **PNI** | | **POS** | | **POT** | | **SEL** | | **SUM** | |
| --- | --- | --- | --- | --- | --- | --- | --- | --- | --- | --- | --- | --- | --- | --- | --- | --- | --- | --- | --- | --- | --- | --- | --- | --- | --- | --- |
| **ALT** |  |  | 0.0018 | 0.0485 | 0.0023 | 0.0490 | 0.0023 | 0.0490 | 0.0017 | 0.0488 | 0.0017 | 0.0485 | 0.0029 | 0.0492 | 0.0026 | 0.0494 | 0.0017 | 0.0508 | 0.0027 | 0.0496 | 0.0020 | 0.0488 | 0.0016 | 0.0484 | 0.0015 | 0.0485 |
| **BAG** | 0.0033 | 0.0720 |  |  | 0.0021 | 0.0706 | 0.0022 | 0.0710 | 0.0057 | 0.0726 | 0.0019 | 0.0705 | 0.0194 | 0.0739 | 0.0025 | 0.0716 | 0.0028 | 0.0715 | 0.0031 | 0.0719 | 0.0042 | 0.0727 | 0.0038 | 0.0723 | 0.0033 | 0.0719 |
| **BOD** | 0.0011 | 0.0277 | 0.0013 | 0.0279 |  |  | 0.0008 | 0.0272 | 0.0006 | 0.0271 | 0.0008 | 0.0271 | 0.0047 | 0.0283 | 0.0015 | 0.0278 | 0.0010 | 0.0277 | 0.0009 | 0.0274 | 0.0008 | 0.0274 | 0.0008 | 0.0273 | 0.0009 | 0.0273 |
| **BRA** | 0.0090 | 0.1487 | 0.0044 | 0.1458 | 0.0044 | 0.1438 |  |  | 0.0042 | 0.1442 | 0.0042 | 0.1452 | 0.0064 | 0.1468 | 0.0042 | 0.1460 | 0.0062 | 0.1478 | 0.0091 | 0.1493 | 0.0063 | 0.1485 | 0.0078 | 0.1501 | 0.0069 | 0.1484 |
| **ILS** | 0.0011 | 0.0276 | 0.0010 | 0.0275 | 0.0006 | 0.0271 | 0.0014 | 0.0280 |  |  | 0.0007 | 0.0271 | 0.0011 | 0.0276 | 0.0015 | 0.0282 | 0.0009 | 0.0277 | 0.0016 | 0.0278 | 0.0017 | 0.0281 | 0.0013 | 0.0280 | 0.0017 | 0.0282 |
| **MOC** | 0.0002 | 0.0038 | 0.0001 | 0.0038 | 0.0001 | 0.0038 | 0.0001 | 0.0038 | 0.0001 | 0.0038 |  |  | 0.0003 | 0.0039 | 0.0002 | 0.0039 | 0.0001 | 0.0038 | 0.0002 | 0.0038 | 0.0001 | 0.0038 | 0.0001 | 0.0038 | 0.0001 | 0.0038 |
| **PAN** | 7.766 | 287.912 | 3.252 | 274.245 | 2.474 | 251.678 | 10.113 | 287.843 | 22.458 | 297.869 | 3.120 | 250.722 |  |  | 18.656 | 299.660 | 2.900 | 267.399 | 8.728 | 285.479 | 6.038 | 286.736 | 5.074 | 279.622 | 32.581 | 298.347 |
| **PNA** | 0.0005 | 0.0125 | 0.0005 | 0.0124 | 0.0004 | 0.0124 | 0.0004 | 0.0123 | 0.0005 | 0.0124 | 0.0006 | 0.0124 | 0.0005 | 0.0124 |  |  | 0.0007 | 0.0125 | 0.0005 | 0.0124 | 0.0006 | 0.0125 | 0.0005 | 0.0124 | 0.0007 | 0.0125 |
| **PNI** | 0.0082 | 0.1639 | 0.0075 | 0.1637 | 0.0068 | 0.1634 | 0.0056 | 0.1617 | 0.0066 | 0.1639 | 0.0060 | 0.1620 | 0.0086 | 0.1644 | 0.0122 | 0.1660 |  |  | 0.0074 | 0.1648 | 0.0069 | 0.1635 | 0.0057 | 0.1632 | 0.0064 | 0.1636 |
| **POS** | 0.0004 | 0.0067 | 0.0002 | 0.0066 | 0.0002 | 0.0066 | 0.0003 | 0.0067 | 0.0002 | 0.0067 | 0.0002 | 0.0067 | 0.0002 | 0.0066 | 0.0003 | 0.0067 | 0.0002 | 0.0066 |  |  | 0.0003 | 0.0067 | 0.0002 | 0.0067 | 0.0004 | 0.0068 |
| **POT** | 0.0001 | 0.0019 | 0.0001 | 0.0019 | 0.0001 | 0.0019 | 0.0001 | 0.0019 | 0.0001 | 0.0019 | 0.0001 | 0.0019 | 0.0001 | 0.0019 | 0.0001 | 0.0019 | 0.0001 | 0.0019 | 0.0001 | 0.0019 |  |  | 0.0001 | 0.0019 | 0.0001 | 0.0019 |
| **SEL** | 0.0073 | 0.1035 | 0.0044 | 0.1027 | 0.0027 | 0.0998 | 0.0041 | 0.1023 | 0.0047 | 0.1035 | 0.0024 | 0.1008 | 0.0043 | 0.1022 | 0.0075 | 0.1040 | 0.0051 | 0.1024 | 0.0060 | 0.1034 | 0.0067 | 0.1038 |  |  | 0.0056 | 0.1036 |
| **SUM** | 0.0007 | 0.0164 | 0.0008 | 0.0163 | 0.0006 | 0.0160 | 0.0007 | 0.0163 | 0.0008 | 0.0164 | 0.0006 | 0.0162 | 0.0005 | 0.0162 | 0.0014 | 0.0166 | 0.0008 | 0.0164 | 0.0010 | 0.0164 | 0.0006 | 0.0163 | 0.0008 | 0.0163 |  |  |
