# Supplementary material for: Coalescent Simulation and Paleodistribution Modeling for Tabebuia rosealba Do Not Support South American Dry Forest Refugia Hypothesis
Source: PLoS One. 2016 Jul 26;11(7):e0159314. doi: 10.1371/journal.pone.0159314 (PMC4961443; doi:10.1371/journal.pone.0159314)
Supplement: S9 Table — (DOCX) [file pone.0159314.s017.docx]

**Coalescent simulation and paleodistribution modeling for *Tabebuia rosealba* do not support South American dry forest refugia hypothesis**

Warita Alves de Melo^1^, Matheus S. Lima-Ribeiro^2^, Levi Carina Terribile^2^, Rosane G. Collevatti^1*^

**S9 Table.** Uncertainty of modeling components on ENM predictions for *Tabebuia roseoalba* as revealed by hierarchical ANOVA. SS: sum of square.

| **Source of variation** | **Median SS** | **Minimun** | **Maximum** |
| --- | --- | --- | --- |
| **TIME** | 0.026 | 0.000 | 0.493 |
| **AOGCM** | 0.212 | 0.016 | 0.809 |
| **ENM** | 0.253 | 0.026 | 0.841 |
| **Residual** | 0.419 | 0.129 | 0.771 |
